# Scp160 deletion suppresses TDP-43 aggregation and toxicity in *Saccharomyces cerevisiae*

**DOI:** 10.15698/mic2026.10.888

**Published:** 2026-10-06

**Authors:** Joan Castells-Ballester, Natalia Shcherbik

**Affiliations:** Department of Cell and Molecular Biology, Rowan-Virtua University, School of Osteopathic Medicine, 2 Medical Center Drive, Stratford, NJ 08084, USA

**Keywords:** TDP-43, Scp160/Vigilin, Bfr1, protein aggregation, proteostasis, proteotoxicity, endoplasmic reticulum, *Saccharomyces cerevisiae*

## Abstract

Protein misfolding and aberrant protein aggregation are central features of neurodegenerative disorders, including amyotrophic lateral sclerosis, where the RNA-binding protein TDP-43 accumulates in abnormal intracellular assemblies. Although yeast models have identified multiple modifiers of TDP-43 toxicity, the cellular factors that determine how aggregation-prone proteins are organized remain incompletely understood. Here, we investigated whether the ribosome- and mRNP-associated proteins Scp160 and Bfr1 influence TDP-43 proteotoxicity and aggregate formation. We report that deletion of *SCP160* or *BFR1* improves growth during TDP43-GFP induction without reducing steady-state reporter abundance and decreases formation of prominent TDP43-GFP foci. Deletion of *BFR1* produced an intermediate TDP-43 phenotype, with a weaker effect than *SCP160* deletion on severe foci formation and toxicity. Importantly, the effect of *SCP160* or *BFR1* deletion on visible protein aggregation was not specific to TDP-43: severe Htt103Q-GFP aggregation was strongly reduced, and formation of VHL-GFP foci was suppressed, including under conditions of proteasome inhibition. Despite these shared effects, the two mutants were not phenotypically identical. Deletion of *SCP160* produced a distinct spatial phenotype in which residual TDP43-GFP foci showed markedly increased association with Sec63-positive ER structures and reduced distance to these regions, whereas *bfr1* Δ cells did not show the same Sec63-associated shift. Together, these findings uncouple TDP43-GFP abundance from toxicity and prominent foci formation and identify Scp160 and Bfr1 as factors that influence the intracellular partitioning of aggregation-prone and misfolded proteins, with an additional role for Scp160 in the spatial organization of TDP43 relative to the ER.

## INTRODUCTION

Protein misfolding and aberrant protein assembly are central features of multiple neurodegenerative disorders, including Alzheimer’s disease, Huntington’s disease, Parkinson’s disease, and amyotrophic lateral sclerosis (ALS) 1, 2. Although these disorders involve different disease-associated proteins and affect distinct neuronal populations, disruption of protein homeostasis and accumulation of misfolded protein species are recurrent features of their pathology 3. The unicellular eukaryote *Saccharomyces cerevisiae* has proven to be a powerful system for studying protein-misfolding disorders because many pathways controlling protein folding, degradation, trafficking, and cellular stress responses are conserved, while yeast genetics enables rapid identification of modifiers of proteotoxicity. Yeast models have reproduced important aspects of the aggregation and toxicity of several disease-associated proteins, including expanded huntingtin, α -synuclein, polyalanine-expanded proteins, TDP-43, and FUS 4–8. The continued utility of yeast for studying neurodegenerative proteinopathies has also been emphasized in recent reviews 9, 10.

A pathological hallmark of ALS and several related disorders is the abnormal accumulation of the RNA-binding protein TDP-43. Under physiological conditions, TDP-43 is predominantly nuclear and participates in multiple aspects of RNA metabolism, including splicing, RNA stability, transport, and translation 11–13. Under pathological conditions, TDP-43 is depleted from the nucleus and accumulates in the cytoplasm, where it forms aberrant assemblies associated with cellular dysfunction and neurodegeneration. Expression of human TDP-43 in yeast recapitulates several key features of this behavior, including cytoplasmic accumulation, formation of fluorescent foci, and dose-dependent toxicity, and has been widely used to identify genetic modifiers of TDP-43 proteotoxicity 5, 10, 14.

Importantly, the relationship between TDP-43 aggregation and toxicity is more complex than a simple increase in aggregate burden. Deep mutational analysis of the TDP-43 prion-like domain showed that variants favoring aggregation could be less toxic, whereas more toxic variants tended to favor smaller and more dynamic assemblies 15. More recently, Park and colleagues showed that shifting TDP-43 toward a more amyloid-like state in an *apt1* Δ *aah1* Δ yeast model reduced toxicity without reducing TDP-43 abundance 16. Additional studies have likewise emphasized that TDP-43 assemblies can differ substantially in their organization and physicochemical properties 17. These findings indicate that the biological consequences of TDP-43 depend not only on how much protein accumulates, but also on the state into which it assembles.

Among yeast RNA-binding proteins, Scp160 is a particularly interesting candidate modifier of TDP-43 proteotoxicity. Scp160 is a multi-KH-domain member of the conserved Vigilin family that associates with polysomes and is enriched at the endoplasmic reticulum, including ER-associated translating ribosomes 18. Rather than functioning as a general translation factor, Scp160 influences the translational efficiency of selected mRNAs 19. More broadly, translating ribosomes provide an important platform for cotranslational folding and protein quality-control pathways that influence nascent-protein fate 20, 21. Scp160 has also been directly implicated in protein aggregation: deletion of *SCP160* reduces aggregation and toxicity of polyglutamine reporters and alters aggregation of multiple endogenous Q/N-rich proteins 22. Bfr1 is a functionally related polysome-associated protein that forms overlapping mRNP complexes with Scp160 and contributes to the translation of selected transcripts, including proteins involved in ER protein quality control 23–25. Together, these observations place Scp160 and Bfr1 at the intersection of RNA organization, selective translation, and protein quality control, raising the possibility that they influence how aggregation-prone proteins are handled within the cell.

Despite these connections, the contribution of Scp160 and its associated mRNP network to TDP-43 proteotoxicity has not been explored. Here, we used a *S. cerevisiae* TDP-43 model to determine whether deletion of *SCP160* or *BFR1* alters TDP-43 abundance, toxicity, and aggregate-like foci formation, whether these effects extend to structurally unrelated misfolded proteins, and whether the residual TDP43-GFP foci differ in their spatial relationship to defined subcellular compartments. Our findings identify Scp160 and Bfr1 as factors that influence the intracellular organization of aggregation-prone and misfolded proteins and reveal a pronounced effect of *SCP160* deletion on the spatial relationship of residual TDP43-GFP foci with Sec63-positive ER regions.

## RESULTS

### Deletion of *SCP160* or *BFR1* suppresses TDP43-GFP toxicity without reducing protein abundance

To determine whether Scp160 and its functionally related partner Bfr1 influence the cellular handling of TDP-43, we expressed human TDP43-GFP from a 2 μ plasmid under control of the inducible *GAL1* promoter 5 in *WT*, *scp160* Δ , and *bfr1* Δ cells. We additionally included *asc1* Δ as an independent comparator because deletion of *ASC1* was recently shown to suppress TDP-43 toxicity and alter TDP43 foci morphology 26, and Asc1 promotes the association of Scp160 with translating ribosomes 27. Because Scp160 and Bfr1 regulate the expression or translational efficiency of selected mRNAs 19, 23, we first asked whether deletion of *SCP160* or *BFR1* altered steady-state TDP43-GFP abundance.

TDP43-GFP expression was induced by transferring cells to galactose-containing medium for 6 h, while parallel cultures maintained in glucose served as a non-induced control. TDP43-GFP was examined by immunoblotting with anti-GFP antibodies, with α -tubulin used as the loading and normalization control. As expected, TDP43-GFP was undetectable or present only at low levels under glucose-repressing conditions, whereas galactose induction produced a strong signal in all four strains (Fig. 1**A**). Quantification of three independent experiments revealed no significant differences in TDP43-GFP abundance among galactose-induced *WT*, *scp160* Δ , *bfr1* Δ , and *asc1* Δ cells (Fig. 1**B**). Thus, the phenotypic differences observed among these strains could not be attributed to reduced steady-state TDP43-GFP abundance.

As a substrate-specific comparison, we examined the secretory protein Rny1-FLAG, previously characterized in our laboratory 28. *RNY1* is among the transcripts associated with Bfr1, which preferentially binds mRNAs encoding secreted and membrane-associated proteins translated at the ER 29, and Bfr1-dependent regulation can produce substrate-specific effects on protein expression 23. Although the major Rny1-FLAG species remained detectable in *WT*, *scp160* Δ , and *bfr1* Δ cells, their relative abundance appeared to differ among the strains (Fig. S1), suggesting that protein abundance can respond to *SCP160* or *BFR1* deletion in a substrate-dependent manner, whereas TDP43-GFP showed no corresponding change.

**Figure 1:**
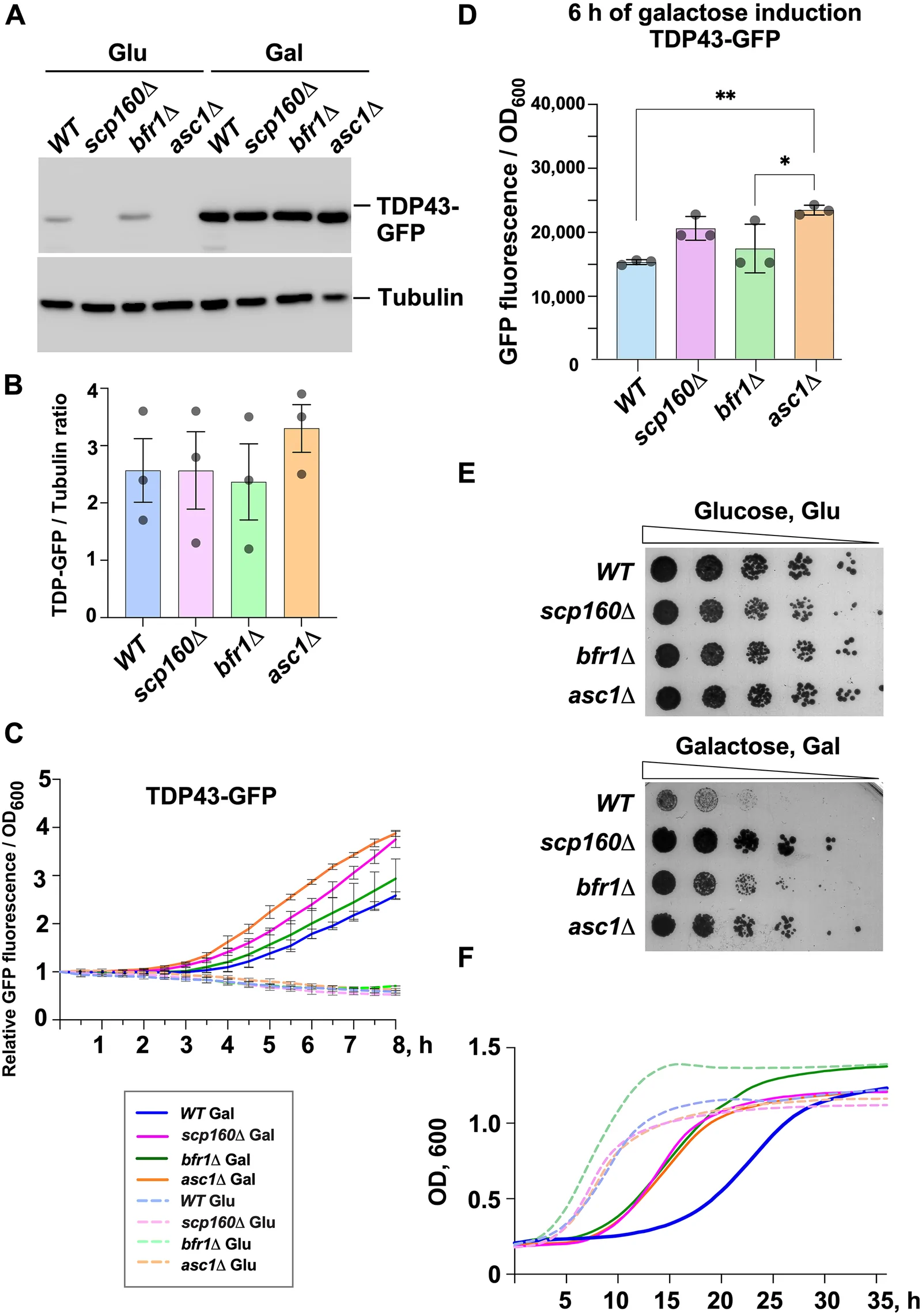
Loss of Scp160, Bfr1, or Asc1 suppresses TDP43-GFP toxicity without reducing reporter abundance. **(A)** Representative immunoblot of TDP43-GFP expression in *WT, scp160* Δ *, bfr1* Δ *,* and *asc1* Δ cells after 6 h under glucose-repressing (Glu) or galactose-inducing (Gal) conditions. TDP43-GFP was detected with anti-GFP antibodies, and α -tubulin served as the loading control. **(B)** Quantification of TDP43-GFP abundance after 6 h of galactose induction. TDP43-GFP signal was normalized to the corresponding tubulin signal within each lane and presented as a GFP/Tubulin ratio on the Y-axis. Symbols represent independent biological experiments; bars show mean ± SD, *n* = 3. No significant differences were detected among strains by ordinary one-way ANOVA followed by Dunnett’s multiple-comparisons test using *WT* as the control. **(C)** Time course of TDP43-GFP fluorescence in cells maintained under glucose-repressing or galactose-inducing conditions. GFP fluorescence was normalized to OD ${}_{600}$ , and values for each culture were expressed relative to the initial measurement. Data are mean ± SD from three independent biological experiments. **(D)** GFP fluorescence normalized to OD ${}_{600}$ after 6 h of galactose induction. Symbols represent independent biological experiments; bars show mean ± SD, *n* = 3. Statistical significance was determined using ordinary one-way ANOVA followed by Tukey’s multiple-comparisons test. The overall effect of genotype was significant, *F*(3, 8) = 8.210, *P* = 0.0080. Tukey-adjusted *P* values were as follows: *WT* versus *scp160* Δ , *P* = 0.0669 (ns); *WT* versus *bfr1* Δ , *P* = 0.6400 (ns); *WT* versus *asc1* Δ , *P* = 0.0074 (^⁎⁎^); *scp160* Δ versus *bfr1* Δ , *P* = 0.3440 (ns); *scp160* Δ versus *asc1* Δ , *P* = 0.4186 (ns); and *bfr1* Δ versus *asc1* Δ , *P* = 0.0372 (^⁎^). ns, not significant; ^⁎^*P* < 0.05; ^⁎⁎^*P* < 0.01. **(E)** Fivefold serial-dilution growth assay of *WT*, *scp160* Δ , *bfr1* Δ , and *asc1* Δ cells carrying the *P* ${}_{GAL1}$ -TDP43-GFP plasmid on SC/Ura- plates containing glucose or galactose. Glucose represses, whereas galactose induces, TDP43-GFP expression. Plates were imaged after 7 days at 30 ${}^{\circ }$ C. **(F)** Growth kinetics of the same strains in liquid SC/Ura- medium containing **either glucose or galactose.**OD ${}_{600}$ was measured every 30 min for 36 h. Solid lines indicate galactose-containing medium, and dashed lines indicate glucose-containing medium. Curves show mean OD ${}_{600}$ values from three independent biological experiments, each measured in technical triplicate. Error bars are omitted for clarity.

To complement this biochemical analysis using an alternative live-cell approach, we next leveraged the fluorescent properties of the TDP43-GFP fusion and measured TDP43-GFP-associated fluorescence in intact cultures. This approach enabled parallel measurements of GFP fluorescence and OD ${}_{600}$ over time, and also avoided a potential technical caveat of incomplete recovery of aggregated or insoluble material during preparation of lysates for immunoblotting. Recorded fluorescence was normalized to the OD ${}_{600}$ value collected at the same time point and expressed relative to the initial measurement (Fig. 1**C**). As expected, cultures maintained under glucose-repressing conditions remained near or below their initial GFP fluorescence/OD ${}_{600}$ values, whereas galactose-induced cultures showed a progressive increase in signal. The fluorescence profiles began to diverge after approximately 4 h of galactose induction, with larger increases observed in *scp160* Δ and *asc1* Δ cultures (Fig. 1**C**).

To compare the strains directly, we analyzed GFP fluorescence/OD ${}_{600}$ after 6 h of galactose induction (Fig. 1**D**). The overall effect of genotype was significant. Pairwise analysis showed that *asc1* Δ cultures had significantly higher GFP fluorescence/OD ${}_{600}$ than both *WT* and *bfr1* Δ cultures, whereas the remaining comparisons did not reach statistical significance. Thus, despite comparable TDP43-GFP protein abundance as determined by immunoblotting (Fig. 1**A, B**), the four strains did not produce identical live-culture fluorescence profiles (Fig. 1**C, D**).

Nevertheless, we did not interpret these differences as evidence for corresponding changes in TDP43-GFP protein abundance. OD ${}_{600}$ provides an indirect estimate of culture density rather than a direct measure of cell number, and its relationship to cell number can be influenced by differences in cell size and morphology. This is particularly relevant because altered cell size or morphology has been reported for *scp160* Δ , *bfr1* Δ , and *asc1* Δ cells 25, 30. We therefore considered that differences in growth and in the relationship between OD ${}_{600}$ and actual cell number could contribute to the live-culture fluorescence readout. This raised the question of whether sustained TDP43-GFP expression affected growth differently among the strains.

To test this possibility directly, we performed a growth assay in which cultures were standardized by direct cell number rather than by OD ${}_{600}$ . Equal numbers (2 × 10 ${}^{6}$ ) of *WT*, *scp160* Δ , *bfr1* Δ , and *asc1* Δ cells carrying *P* ${}_{GAL1}$ -TDP43-GFP were subjected to serial dilution and spotted onto glucose- or galactose-containing agar. All four strains grew comparably under glucose-repressing conditions, indicating that the deletions did not substantially impair growth in the absence of TDP43-GFP induction. In contrast, galactose-induced TDP43-GFP expression strongly inhibited growth of *WT* cells, whereas *scp160* Δ , *bfr1* Δ , and *asc1* Δ cells showed improved growth (Fig. 1**E**). Growth improvement was most pronounced in *scp160* Δ and *asc1* Δ cells, while *bfr1* Δ cells displayed a weaker but reproducible improvement. Importantly, the protection observed in *asc1* Δ cells is consistent with the independently reported suppression of TDP-43 toxicity by *ASC1* deletion in yeast 26.

Growth kinetics in liquid culture confirmed the phenotype observed in the spot assay (Fig. 1**F**). Under glucose-repressing conditions, all four strains displayed robust growth. Following TDP43-GFP induction, however, *WT* cultures exhibited a pronounced growth delay, whereas *scp160* Δ , *bfr1* Δ , and *asc1* Δ cultures grew substantially better under the same conditions.

Taken together, these results demonstrate that suppression of TDP43-GFP toxicity in the deletion strains occurs despite comparable steady-state protein abundance, prompting us to examine whether TDP43-GFP aggregate-like foci formation differs among the strains.

### Deletion of *SCP160*, *BFR1*, or *ASC1* reduces TDP43-GFP aggregation, with the strongest suppression of severe aggregates in *scp160*Δ and *asc1*Δ cells

Because protection from TDP-43 toxicity could not be explained by reduced TDP43-GFP abundance, we next asked whether it was accompanied by changes in TDP43-GFP aggregation. Genetic suppression of TDP-43 toxicity can occur without reduced expression while substantially altering inclusion morphology 14, and the relationship between TDP-43 aggregation and toxicity depends on assembly properties rather than aggregate abundance alone 15. Most directly relevant, deletion of *ASC1* was recently shown to suppress TDP-43 toxicity and produce smaller TDP43 foci in yeast 26. We therefore examined TDP43-GFP fluorescence patterns in *WT*, *scp160* Δ , *bfr1* Δ , and *asc1* Δ cells after 6 h of galactose induction.

We found that in *WT* cells, TDP43-GFP frequently accumulated in bright punctate foci, consistent with its established aggregation-prone phenotype in yeast 5, 14, 31. In contrast, all three deletion strains contained fewer cells with prominent foci and more cells displaying diffuse or undetectable GFP fluorescence (Fig. 2**A**). Cells were classified as having no detectable GFP, diffuse GFP, moderate aggregates, or severe aggregates based on the fluorescence patterns shown in Fig. 2**B**. Approximately one-third of *WT* cells contained visible TDP43-GFP aggregates, compared with approximately one-fifth of cells in each deletion strain (Fig. 2**C**).

A stronger distinction emerged when severe aggregates were analyzed separately. Relative to *WT*, the severe-aggregate population was reduced nearly eightfold in *scp160* Δ and approximately fivefold in *asc1* Δ cells (Fig. 2**D**). The reduction in *bfr1* Δ cells was more modest, and severe aggregates remained significantly more frequent than in either *scp160* Δ or *asc1* Δ . Thus, although all three deletions reduced overall TDP43-GFP aggregation, deletion of *SCP160* or *ASC1* produced a particularly strong shift away from the severe-aggregate phenotype, consistent with the smaller TDP43 foci previously reported in *asc1* Δ cells 26.

**Figure 2:**
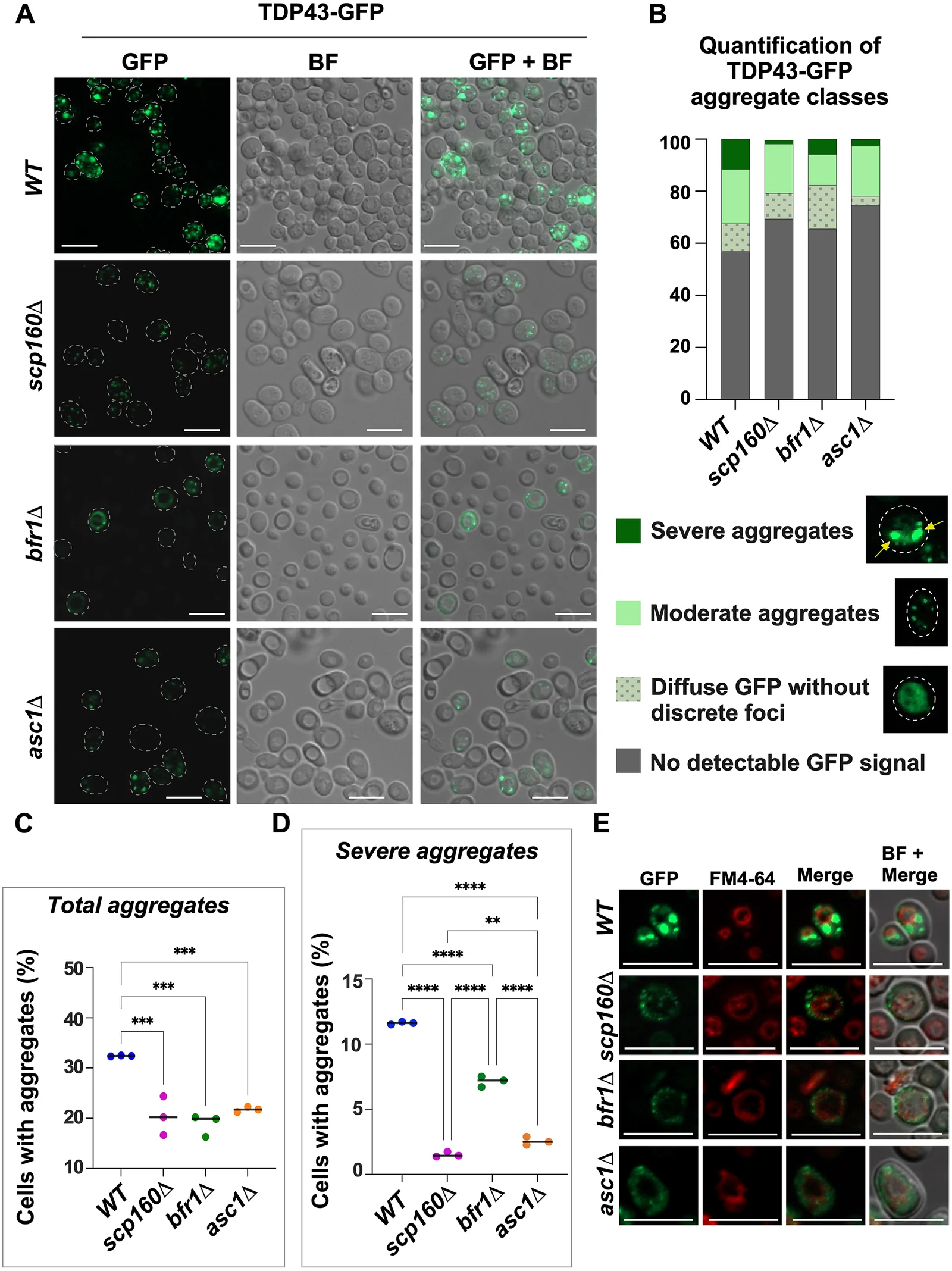
Deletion of *SCP160*, *BFR1*, or *ASC1* reduces TDP43-GFP aggregation, with the strongest suppression of severe aggregates in *scp160* Δ and *asc1* Δ cells. **(A)** Representative fluorescence microscopy images of *WT*, *scp160* Δ , *bfr1* Δ , and *asc1* Δ cells expressing *P* ${}_{GAL1}$ -TDP43-GFP. Cells were grown in synthetic glucose medium lacking uracil to mid-log phase and shifted to galactose-containing medium for 6 h to induce TDP43-GFP expression. GFP, bright-field (BF), and GFP + BF overlay images are shown. Dashed white outlines in the GFP images indicate cell boundaries. Scale bars, 10 μ m. **(B)** Quantification of TDP43-GFP aggregate classes after 6 h of galactose induction. Cells were classified as having no detectable GFP signal, diffuse GFP without discrete foci, moderate aggregates, or severe aggregates. Moderate aggregates were defined as one or more small TDP43-GFP puncta, whereas severe aggregates were defined as one large bright foci and/or multiple prominent foci within the same cell. Stacked bars show the distribution of scored cells among the four categories. Representative examples of diffuse GFP, moderate aggregates, and severe aggregates are shown below. Yellow arrows indicate prominent foci in the severe-aggregate example. **(C)** Percentage of cells containing visible TDP43-GFP aggregates. Total aggregate-positive cells were defined as cells containing either moderate or severe aggregates. Each dot represents an independent biological experiment, and horizontal lines indicate the mean. Statistical significance was determined using ordinary one-way ANOVA followed by Tukey’s multiple-comparisons test. The overall effect of genotype was significant, F(3, 8) = 22.95, *P* = 0.0003. Tukey-adjusted *P* values were as follows: *WT* versus *scp160* Δ , *P* = 0.0008 (^⁎⁎⁎^); *WT* versus *bfr1* Δ , *P* = 0.0003 (^⁎⁎⁎^); *WT* versus *asc1* Δ , *P* = 0.0017 (^⁎⁎^); *scp160* Δ versus *bfr1* Δ , *P* = 0.8064 (ns); *scp160* Δ versus *asc1* Δ , *P* = 0.8834 (ns); and *bfr1* Δ versus *asc1* Δ , *P* = 0.4176 (ns). Three independent biological experiments were analyzed for each strain. **(D)** Percentage of cells containing severe TDP43-GFP aggregates. Each dot represents an independent biological experiment, and horizontal lines indicate the mean. Statistical significance was determined using ordinary one-way ANOVA followed by Tukey’s multiple-comparisons test. The overall effect of genotype was significant, F(3, 8) = 824.6, *P* < 0.0001. Tukey-adjusted *P* values were as follows: *WT* versus *scp160* Δ , *P* < 0.0001 (^⁎⁎⁎⁎^); *WT* versus *bfr1* Δ , *P* < 0.0001 (^⁎⁎⁎⁎^); *WT* versus *asc1* Δ , *P* < 0.0001 (^⁎⁎⁎⁎^); *scp160* Δ versus *bfr1* Δ , *P* < 0.0001 (^⁎⁎⁎⁎^); *scp160* Δ versus *asc1* Δ , *P* = 0.0080 (^⁎⁎^); and *bfr1* Δ versus *asc1* Δ , *P* < 0.0001 (^⁎⁎⁎⁎^). Three independent biological experiments were analyzed for each strain. **(E)** Representative enlarged images of *WT*, *scp160* Δ , *bfr1* Δ , and *asc1* Δ cells stained with FM4-64 to visualize the vacuolar membrane. GFP + FM4-64, GFP, FM4-64, and bright-field (BF) + GFP + FM4-64 images are shown. FM4-64 staining identified the central GFP-negative compartment as the vacuole, indicating that the apparent ring-like TDP43-GFP pattern reflects cytoplasmic fluorescence surrounding the vacuole. Scale bars, 10 μ m.

The reduction in discrete foci was not accompanied by a uniform loss of GFP fluorescence, consistent with the immunoblot analysis showing comparable steady-state TDP43-GFP abundance (Fig. 1**A–B**). In *bfr1* Δ cells, diffuse fluorescence frequently appeared as a ring surrounding a central GFP-negative region. FM4-64 staining identified this region as the vacuole, indicating that the apparent ring reflected TDP43-GFP surrounding the vacuolar compartment (Fig. 2**E**).

Taken together, deletion of *SCP160*, *BFR1*, or *ASC1* reduced visible TDP43-GFP aggregation without reducing reporter abundance, with the strongest suppression of severe aggregates in *scp160* Δ and *asc1* Δ cells. This pattern qualitatively paralleled the relative protection from TDP-43 toxicity observed in the growth assays (Fig. 1**E–F**), but does not by itself establish a causal relationship between severe aggregates and toxicity.

### Formation of severe Htt103Q-GFP aggregates is suppressed in *scp160* Δ , *bfr1* Δ , and *asc1* Δ cells

The reduction in prominent TDP43-GFP foci prompted us to ask whether this phenotype was specific to TDP-43 or extended to another aggregation-prone substrate. We therefore examined Htt103Q-GFP, a polyglutamine-expanded huntingtin fragment that forms highly insoluble intracellular aggregates in yeast 7, 32. Scp160 has previously been implicated in polyQ aggregation, with *SCP160* deletion reducing aggregation of polyQ reporters and endogenous Q/N-rich proteins 22, and an independent screen identified *SCP160*, *BFR1*, and *ASC1* among genes influencing polyQ-induced growth inhibition 33.

Htt103Q-GFP was expressed from the *GAL1* promoter in *WT*, *scp160* Δ , *bfr1* Δ , and *asc1* Δ cells for 6 h. In *WT* cells, Htt103Q-GFP typically formed a single large, bright inclusion, consistent with the established behavior of expanded huntingtin fragments in yeast 7, 32 (Fig. 3**A**). In contrast, the deletion strains contained predominantly cells with diffuse GFP fluorescence, with smaller puncta present in a subset of cells (Fig. 3**A–B**).

Quantification revealed that severe Htt103Q-GFP inclusions were present in approximately 9-10% of *WT* cells but were nearly eliminated in *scp160* Δ cells and strongly reduced in *bfr1* Δ cells (Fig. 3**C**). *asc1* Δ cells also showed a substantial reduction, although the remaining severe-aggregate fraction was greater than in *scp160* Δ . The *bfr1* Δ phenotype is particularly notable because a previous genome-wide loss-of-function screen identified *BFR1* deletion as a suppressor of Htt103Q-mediated toxicity but did not establish that this protection was accompanied by reduced Htt103Q aggregation 34. Our data therefore extend this observation by linking *BFR1* deletion to a substantial reduction in severe Htt103Q-GFP aggregates.

**Figure 3:**
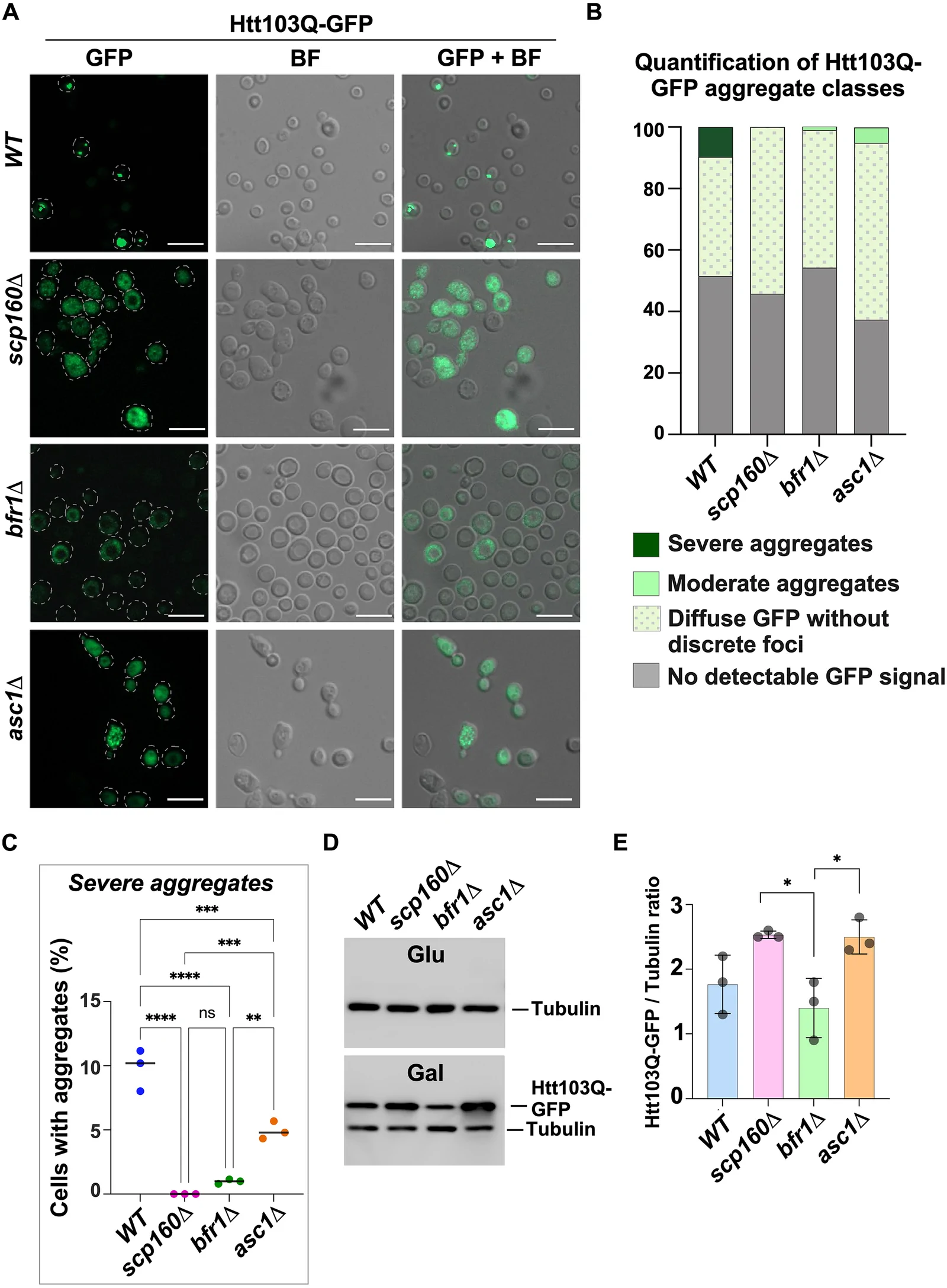
Loss of Scp160, Bfr1, or Asc1 suppresses severe Htt103Q-GFP aggregation without a corresponding reduction in reporter abundance. **(A)** Representative fluorescence microscopy images of *WT*, *scp160* Δ , *bfr1* Δ , and *asc1* Δ cells expressing *P* ${}_{GAL1}$ -Htt103Q-GFP. Cells were grown to mid-log phase in synthetic glucose medium lacking uracil and shifted to galactose-containing medium to induce Htt103Q-GFP expression. GFP, bright-field (BF), and GFP + BF overlay images are shown. Dashed white outlines in the GFP images indicate cell boundaries. Scale bars, 10 μ m. **(B)** Quantification of Htt103Q-GFP localization. As in Figure 2, cells were classified as having no detectable GFP signal, diffuse GFP fluorescence without discrete foci, moderate aggregates, or severe aggregates. Moderate aggregates were defined as one or more small GFP puncta, whereas severe aggregates were defined as one or more large, prominent GFP foci. Cells containing both small and large foci were assigned to the severe-aggregate category. Stacked bars show the distribution of scored cells among the four categories. **(C)** Percentage of cells containing severe Htt103Q-GFP aggregates. Each dot represents an independent biological experiment, and horizontal lines indicate the mean. Statistical significance was determined using ordinary one-way ANOVA followed by Tukey’s multiple-comparisons test. The overall effect of genotype was significant, F(3, 8) = 76.63, *P* < 0.0001. Tukey-adjusted *P* values were as follows: *WT* versus *scp160* Δ , *P* < 0.0001 (^⁎⁎⁎⁎^); *WT* versus *bfr1* Δ , *P* < 0.0001 (^⁎⁎⁎⁎^); *WT* versus *asc1* Δ , *P* = 0.0007 (^⁎⁎⁎^); *scp160* Δ versus *bfr1* Δ , *P* = 0.5559 (ns); *scp160* Δ versus *asc1* Δ , *P* = 0.0006 (^⁎⁎⁎^); and *bfr1* Δ versus *asc1* Δ , *P* = 0.0025 (^⁎⁎^). **(D)** Representative immunoblot of Htt103Q-GFP expression in *WT*, *scp160* Δ , *bfr1* Δ , and *asc1* Δ cells under glucose-repressing (Glu) or galactose-inducing (Gal) conditions. Htt103Q-GFP was detected using anti-GFP antibodies, and α -tubulin served as the loading and normalization control. **(E)** Quantification of Htt103Q-GFP abundance in galactose-induced cells from three independent biological experiments. Htt103Q-GFP signal was normalized to the corresponding tubulin signal within each lane and presented as a GFP/Tubulin ratio on the Y-axis. Symbols represent independent biological experiments; bars show mean ± SD, *n* = 3. The overall effect of genotype was significant by ordinary one-way ANOVA, F(3,8) = 7.717, *P* = 0.0095. Tukey’s multiple-comparisons test detected no significant differences between *WT* and *scp160* Δ (adjusted *P* = 0.1027), *WT* and *bfr1* Δ (*P* = 0.5947), or *WT* and *asc1* Δ (*P* = 0.1211). Htt103Q-GFP abundance was significantly higher in *scp160* Δ than in *bfr1* Δ cells (adjusted *P* = 0.0171) and in *asc1* Δ than in *bfr1* Δ cells (adjusted *P* = 0.0200); *scp160* Δ and *asc1* Δ did not differ significantly (*P* = 0.9994). ns, not significant; ^⁎^*P* < 0.05.

**Figure 4:**
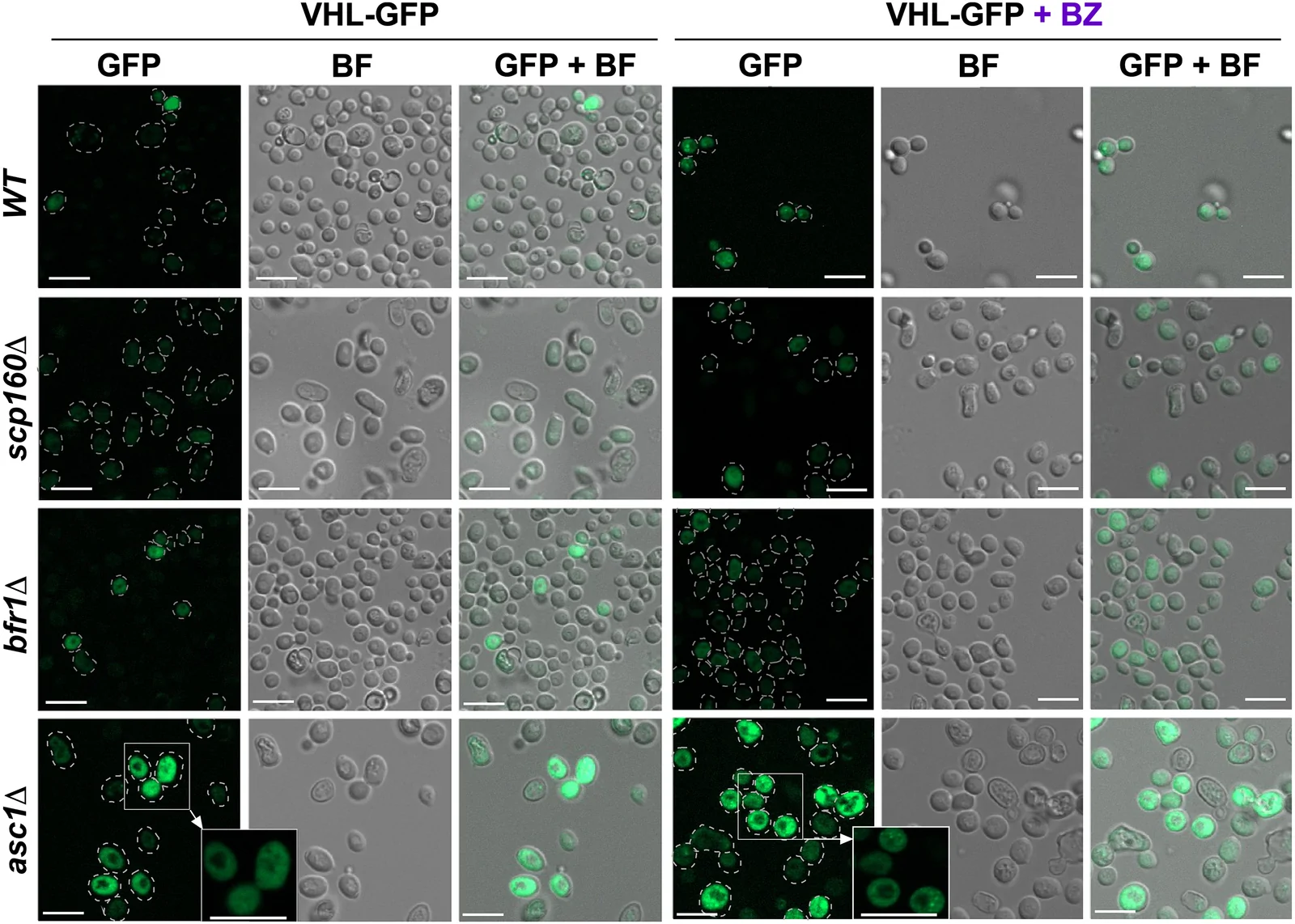
The soluble misfolded-protein reporter VHL-GFP remains predominantly diffuse in *scp160* Δ , *bfr1* Δ , and *asc1* Δ cells. Representative fluorescence microscopy images of *WT*, *scp160* Δ *, bfr1* Δ *,* and *asc1* Δ cells expressing *P* ${}_{\mathrm{GAL1}}$ -VHL-GFP in the absence or presence of the proteasome inhibitor bortezomib (BZ). VHL-GFP expression was induced in galactose-containing medium for 5 h, after which cultures were treated with DMSO (-BZ) or with 50 μ M BZ for an additional 1 h before imaging. GFP fluorescence, bright-field images (BF), and GFP + BF overlays are shown. Dashed white outlines indicate cell boundaries identified from the corresponding BF images. Boxed regions in the *asc1* Δ GFP panels are shown at higher magnification in the insets using a shorter GFP exposure time to minimize saturation and better visualize VHL-GFP distribution. Scale bars, 10 μ m.

Furthermore, immunoblotting confirmed Htt103Q-GFP production in all four strains (Fig. 3**D**), and although some strain-dependent variation was detected, none of the mutants differed significantly from *WT* (Fig. 3**E**). In particular, *scp160* Δ cells accumulated abundant Htt103Q-GFP while almost completely lacking severe inclusions (Fig. 3**C–E**).

These findings demonstrate that the effect of *SCP160*, *BFR1*, and *ASC1* deletion on prominent aggregate formation is not restricted to TDP-43. However, both TDP43-GFP and Htt103Q-GFP are strongly aggregation-prone proteins. We therefore next examined VHL-GFP, a structurally unrelated and comparatively soluble misfolded quality-control substrate whose abundance and intracellular partitioning are strongly influenced by proteasomal degradation 35.

### Deletion of *SCP160*, *BFR1*, or *ASC1* suppresses VHL-GFP foci formation, with a distinct effect of Asc1 on reporter abundance

Unlike TDP43-GFP and Htt103Q-GFP, VHL-GFP is a folding-defective quality-control substrate that remains comparatively soluble and is normally targeted for proteasomal degradation. In yeast, VHL cannot adopt its native conformation in the absence of its mammalian binding partners elongin B and C and remains associated with cytosolic chaperone pathways before degradation by the ubiquitin-proteasome system 36, 37. When degradation becomes limiting, misfolded VHL can concentrate in organized quality-control compartments 35, 38. VHL also partitions differently from polyQ-expanded huntingtin and remains substantially more dynamic 35, providing a complementary misfolded substrate.

*WT*, *scp160* Δ , *bfr1* Δ , and *asc1* Δ cells expressing VHL-GFP from *P* ${}_{\mathrm{GAL1}}$ were induced in galactose-containing medium for 5 h and then incubated for an additional 1 h in the absence or presence of the proteasome inhibitor bortezomib (BZ). Proteasome inhibition was included in this experiment to reduce degradation of folding-defective VHL and promote its accumulation in visible quality-control assemblies, an approach previously established for analysis of intracellular sequestration of misfolded VHL and related quality-control substrates 39.

Our microscopy analysis, followed by quantification, revealed that in BZ-untreated *WT* cells, VHL-GFP was detectable in only a subset of cells, and approximately 24% of GFP-positive cells contained moderate or severe foci. BZ treatment increased detectable VHL-GFP fluorescence and raised the foci-positive population to approximately 42% (Figs. 4 and 5**A–B**), consistent with the established behavior of VHL when proteasomal degradation becomes limiting 35, 36, 40.

In contrast, VHL-GFP remained predominantly diffuse in *scp160* Δ and *bfr1* Δ cells under both untreated and BZ-treated conditions (Fig. 4). Moderate or severe foci were nearly absent before treatment, and proteasome inhibition produced little or no increase in the foci-positive population (Figs. 4 and 5**A–B**). Because GFP fluorescence remained readily detectable, suppression of VHL-GFP foci could not be explained by loss of reporter expression. *asc1* Δ cells showed similarly strong suppression of VHL-GFP foci but were distinguished by unusually intense, predominantly diffuse fluorescence (Fig. 4). Immunoblotting confirmed a dramatic increase in VHL-GFP protein abundance, reaching approximately 100-fold above *WT* in BZ-untreated cells and approximately 60-fold following BZ treatment, whereas reporter levels in *WT*, *scp160* Δ , and *bfr1* Δ remained comparable (Fig. 5**C–D**). Thus, even markedly increased VHL-GFP abundance was insufficient to promote efficient foci formation in *asc1* Δ cells.

Together, these results extend the phenotype observed with TDP43-GFP and Htt103Q-GFP to a structurally and biochemically distinct misfolded substrate. Deletion of *SCP160*, *BFR1*, or *ASC1* strongly reduced VHL-GFP foci formation, supporting a broader role for these factors in determining whether misfolded proteins remain relatively diffuse or become concentrated into visible intracellular structures.

### TDP43-GFP foci lack detectable ThT-specific reactivity

Having established that deletion of *SCP160* or *BFR1* alters visible aggregate formation across several misfolded-protein reporters, we next focused on these two mutants to further characterize the residual TDP43 foci. TDP-43 can populate assemblies with different structural properties depending on cellular and experimental context 15, 41, 42. We therefore asked whether the altered TDP43 foci observed in *scp160* Δ and *bfr1* Δ cells showed detectable Thioflavin T (ThT) reactivity indicative of amyloid-like organization.

Purified full-length TDP-43 has been reported to form aggregates lacking detectable ThT or Congo Red reactivity, although amyloid-like TDP-43 structures can form under other experimental conditions 31, 43. For this analysis, we used TDP43-YFP so that reporter fluorescence and ThT-associated fluorescence could be monitored separately in the YFP and CFP channels, an approach previously used to examine ThT staining of TDP43-YFP foci in yeast 41. Htt103Q-YFP served as a positive control because its yeast inclusions are detergent-resistant and ThT-reactive 44. As expected, strong ThT-channel fluorescence was associated with Htt103Q-YFP inclusions (Fig. 6**A**; Fig. S2).

**Figure 5:**
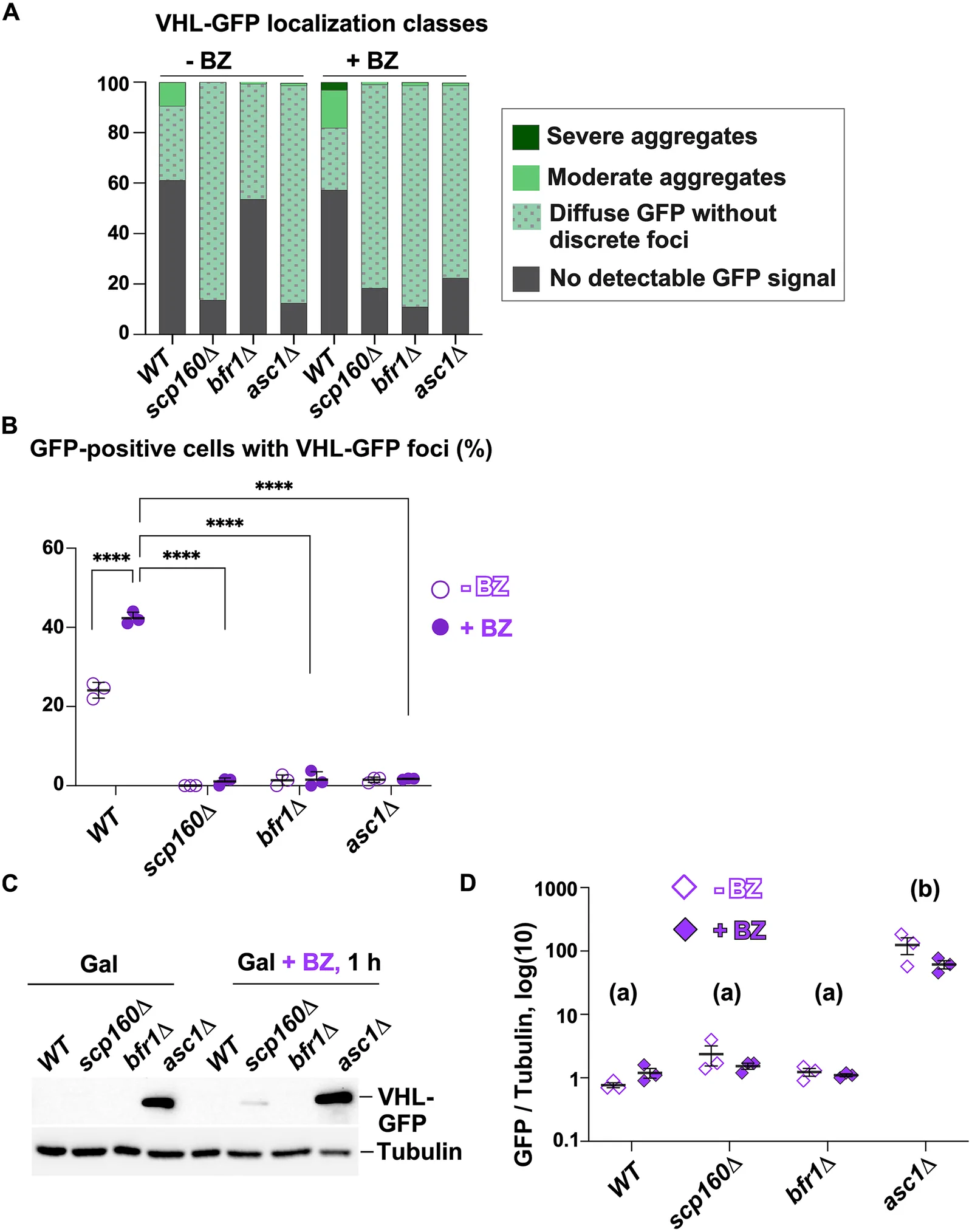
Scp160, Bfr1, and Asc1 promote VHL-GFP foci formation, with a distinct effect of Asc1 on reporter abundance. **(A)** Distribution of cells among four mutually exclusive VHL-GFP localization classes: no detectable GFP signal, diffuse GFP fluorescence without discrete foci, moderate foci, or severe foci (from Figure 4). Moderate foci were defined as one or more small GFP puncta, whereas severe foci were defined as one large, bright GFP foci and/or multiple prominent foci within the same cell. Cells containing both small and large foci were assigned to the severe-foci category. Stacked bars show the pooled percentage of cells in each category across three independent biological experiments. **(B)** Percentage of GFP-positive cells containing moderate or severe VHL-GFP foci. Cells lacking detectable GFP signal were excluded from this calculation. Individual points represent independent biological experiments; horizontal lines and error bars indicate mean ± SD, *n* = 3. Open circles indicate vehicle-treated cultures and filled circles indicate BZ-treated cultures. Data were analyzed by ordinary two-way ANOVA with genotype and BZ treatment as factors. Significant effects were detected for genotype, F(3,16) = 932.4, *P* < 0.0001; BZ treatment, F(1,16) = 87.81, *P* < 0.0001; and the genotype × BZ interaction, F(3,16) = 71.56, *P* < 0.0001. BZ-treated and vehicle-treated cultures were compared within each genotype using Šídák’s multiple-comparisons test. BZ significantly increased VHL-GFP foci formation in *WT* cells (adjusted *P* < 0.0001, ^⁎⁎⁎⁎^), but not in *scp160* Δ (adjusted *P* = 0.7985, ns), *bfr1* Δ (adjusted *P* = 0.9996, ns), or *asc1* Δ (adjusted *P* = 0.9995, ns). **(C)** Representative immunoblot of VHL-GFP expression in *WT*, *scp160* Δ , *bfr1* Δ , and *asc1* Δ cells grown under VHL-GFP-inducing conditions in the absence or presence of BZ. Cells were grown and BZ-treated as in (A), and cell extracts were analyzed by western blotting using anti-GFP and anti-tubulin antibodies. **(D)** Quantification of VHL-GFP abundance in galactose-induced cells from three independent biological experiments. VHL-GFP signal was normalized to the corresponding tubulin signal within each lane and presented as a GFP/Tubulin ratio on the Y-axis, displayed on a logarithmic base-10 scale. Each point represents an independent biological experiment; horizontal lines and error bars indicate mean ± SD, *n* = 3. Open symbols indicate DMSO-treated cultures and filled symbols indicate BZ-treated cultures. Data were analyzed using ordinary two-way ANOVA with genotype and BZ treatment as factors, including the genotype × treatment interaction. A significant effect of genotype was detected, F(3,16) = 23.63, *P* < 0.0001, whereas the effect of BZ treatment, F(1,16) = 2.856, *P* = 0.1104, and the genotype × treatment interaction, F(3,16) = 2.793, *P* = 0.0740, were not significant. Genotypes were compared within each treatment condition using Tukey’s multiple-comparisons test. Under galactose induction without BZ, VHL-GFP abundance was greater in *asc1* Δ than in *WT*, *scp160* Δ , or *bfr1* Δ cells (all adjusted *P* < 0.0001). In the presence of BZ, VHL-GFP abundance remained greater in *asc1* Δ than in *WT* (*P* = 0.0261), *scp160* Δ (*P* = 0.0270), or *bfr1* Δ (*P* = 0.0258). No significant differences were detected among *WT*, *scp160* Δ , and *bfr1* Δ cells under either condition. Groups marked “a” were not significantly different, whereas groups assigned “b” differed significantly within the same treatment condition based on Tukey’s multiple-comparisons test with adjusted *P* < 0.05.

TDP43-YFP showed a different pattern. Although local CFP-channel fluorescence was detected at or near some TDP43-YFP foci after ThT staining, comparable signal was also present in samples not exposed to ThT. Quantification of CFP-channel intensity at YFP-defined foci relative to the surrounding cellular signal showed no increase following ThT addition in *WT*, *scp160* Δ , or *bfr1* Δ cells and no genotype-dependent differences (Fig. 6**B**). Thus, under the conditions examined, TDP43-YFP foci in all three strains lacked detectable ThT-specific enrichment, consistent with previous observations of ThT-negative full-length TDP-43 aggregates and yeast TDP43 foci 31, 41.

**Figure 6:**
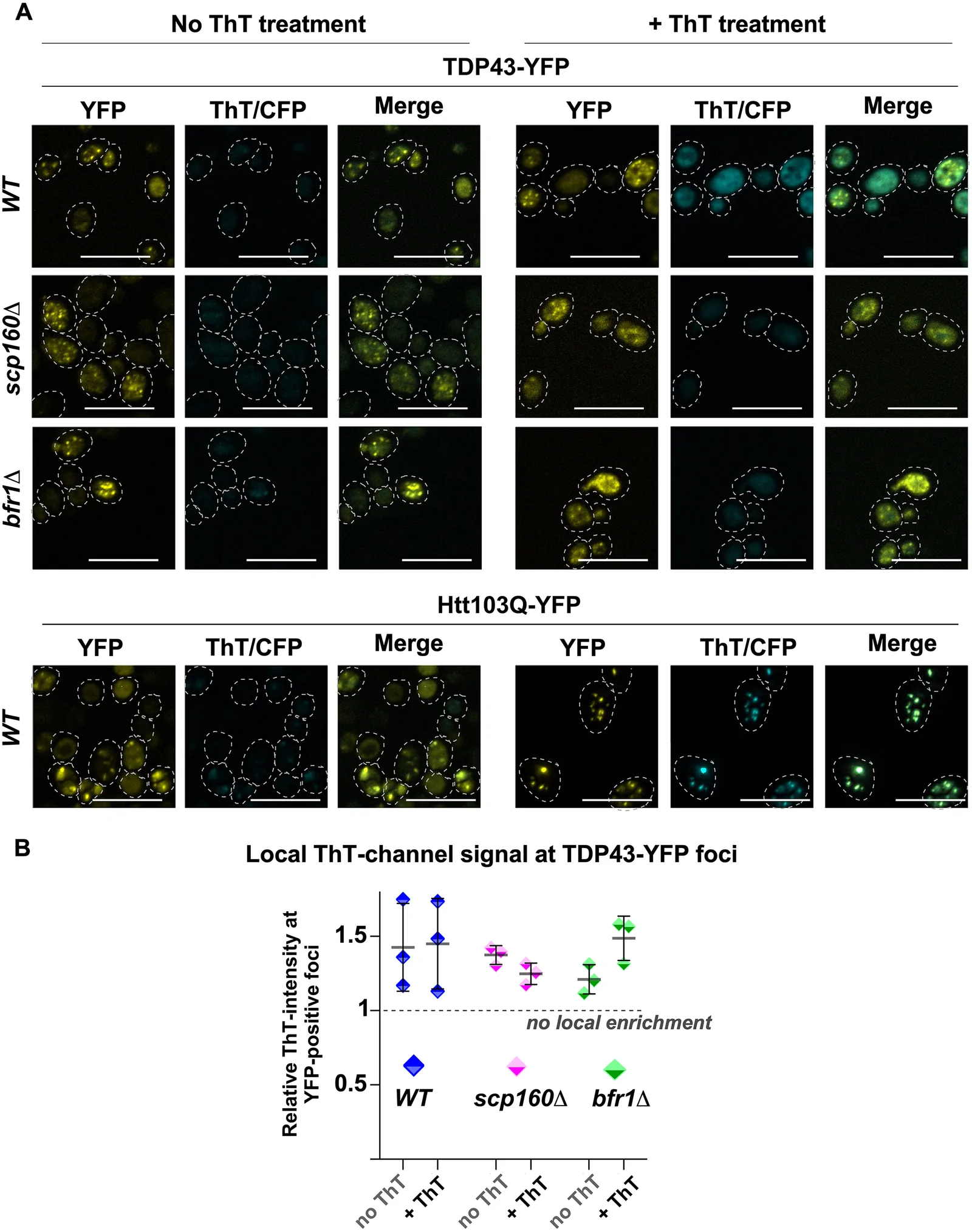
TDP43-YFP foci do not show detectable specific enrichment of Thioflavin T in *WT, scp160* Δ , or *bfr1* Δ cells. **(A)** Representative fluorescence microscopy images of *WT*, *scp160* Δ *,* and *bfr1* Δ cells expressing TDP43-YFP in the absence or presence of Thioflavin T (ThT). TDP43-YFP was detected in the YFP channel, and ThT-associated fluorescence was recorded in the CFP channel. Before imaging, cells were stained with 50 μ g/mL ThT for 20 min. Corresponding YFP, ThT/CFP, and merged images are shown. Dashed outlines indicate cell boundaries. *WT* cells expressing Htt103Q-YFP are shown as a positive control; Htt103Q-YFP inclusions were accompanied by prominent focal ThT-channel fluorescence. Extended image sets showing YFP, ThT/CFP, Merge, Merge + BF, and BF are provided in Fig. S2. **(B)** Quantification of local ThT-channel signal at TDP43-YFP foci. TDP43-YFP foci were identified from the YFP channel, and mean CFP-channel intensity within each YFP-defined foci was normalized to the CFP-channel intensity in the surrounding local reference region. A ratio of 1.0 therefore represents no local enrichment of CFP-channel signal at the YFP foci. For each independent culture, foci-level measurements were summarized to yield a single value. Data are shown as individual biological replicates with mean ± SD; *n* = 3 independent cultures per genotype and treatment condition. Statistical significance was assessed by ordinary two-way ANOVA with genotype and ThT treatment as factors. No significant genotype × ThT treatment interaction was detected, F(2,12) = 1.690, *P* = 0.2256, and neither the main effect of ThT treatment, F(1,12) = 0.4120, *P* = 0.5330, nor genotype, F(2,12) = 0.6918, *P* = 0.5196, was significant. Tukey’s multiple-comparisons test detected no significant genotype differences within either treatment condition: without ThT, *WT* versus *scp160* Δ , adjusted *P* = 0.9429; *WT* versus *bfr1* Δ *, P* = 0.3844; *scp160* Δ versus *bfr1* Δ *, P* = 0.5635; with ThT, *WT* versus *scp160* Δ *, P* = 0.4259; *WT* versus *bfr1* Δ *, P* = 0.9701; *scp160* Δ versus *bfr1* Δ *, P* = 0.3134.

Together, these results distinguish the residual TDP43 foci from the ThT-reactive Htt103Q inclusions and indicate that the altered TDP43 morphology observed in *scp160* Δ and *bfr1* Δ cells is not accompanied by detectable acquisition of ThT-reactive amyloid-like organization.

### Deletion of *SCP160* alters the spatial relationship of TDP43-GFP foci with Sec63-positive ER structures

To extend our characterization beyond TDP43-GFP morphology and assembly properties, we next examined the subcellular context of the residual TDP43-GFP foci. Spatial partitioning is an important component of protein quality control, with misfolded proteins directed to defined intracellular sites rather than accumulating randomly 35, 45, 46. We therefore asked whether deletion of *SCP160* or *BFR1* altered the spatial relationship of TDP43-GFP foci with selected cellular compartments implicated in TDP-43 biology.

Because mitochondrial association has been implicated in TDP-43 toxicity in yeast and mammalian systems 47–49, we first examined TDP43-GFP relative to MitoTracker Red-positive regions. TDP43-GFP showed modest enrichment at mitochondrial regions in *WT*, *scp160* Δ , and *bfr1* Δ cells, consistent with previous reports of perimitochondrial TDP-43 foci in yeast and partial mitochondrial localization in neuronal models 47–49; however, the extent of this association did not differ significantly among the strains (Fig. S3).

We next examined Atg8-positive structures because autophagic and endosomal-vacuolar pathways contribute to TDP-43 turnover in yeast 50, 51, and suppression of TDP-43 toxicity in *asc1* Δ cells has recently been linked to enhanced noncanonical autophagy 26. A subset of TDP43-GFP foci overlapped Atg8-mCherry-positive structures, but the frequency of this association was comparable in *WT*, *scp160* Δ , and *bfr1* Δ cells (Fig. S4).

We also examined TDP43-GFP foci relative to Nup49-mCherry-positive nuclear-pore structures because perinuclear TDP-43 foci have been described in yeast, while TDP-43 pathology in mammalian systems can disrupt nuclear-pore components and nucleocytoplasmic transport 47, 52. A subset of TDP43-GFP foci directly overlapped Nup49-positive structures in all three strains (Fig. 7**A**, Fig. S5), but neither direct overlap nor the fraction of foci located within 0.5 μ m of Nup49-positive structures differed significantly among genotypes (Fig. 7**B–C**).

A markedly different pattern emerged when we examined the ER using Sec63-RFP (Fig. 7**D**; Fig. S6). The ER was of particular interest because TDP-43 assemblies associated with ER-exit sites have recently been described in mammalian cells 42, while Scp160 localizes to the nuclear envelope and ER and associates with ER-bound polysomes 18, 53. Bfr1 also has ER-linked functions, including regulation of Pmt1 and Pmt2 translation 23.

**Figure 7:**
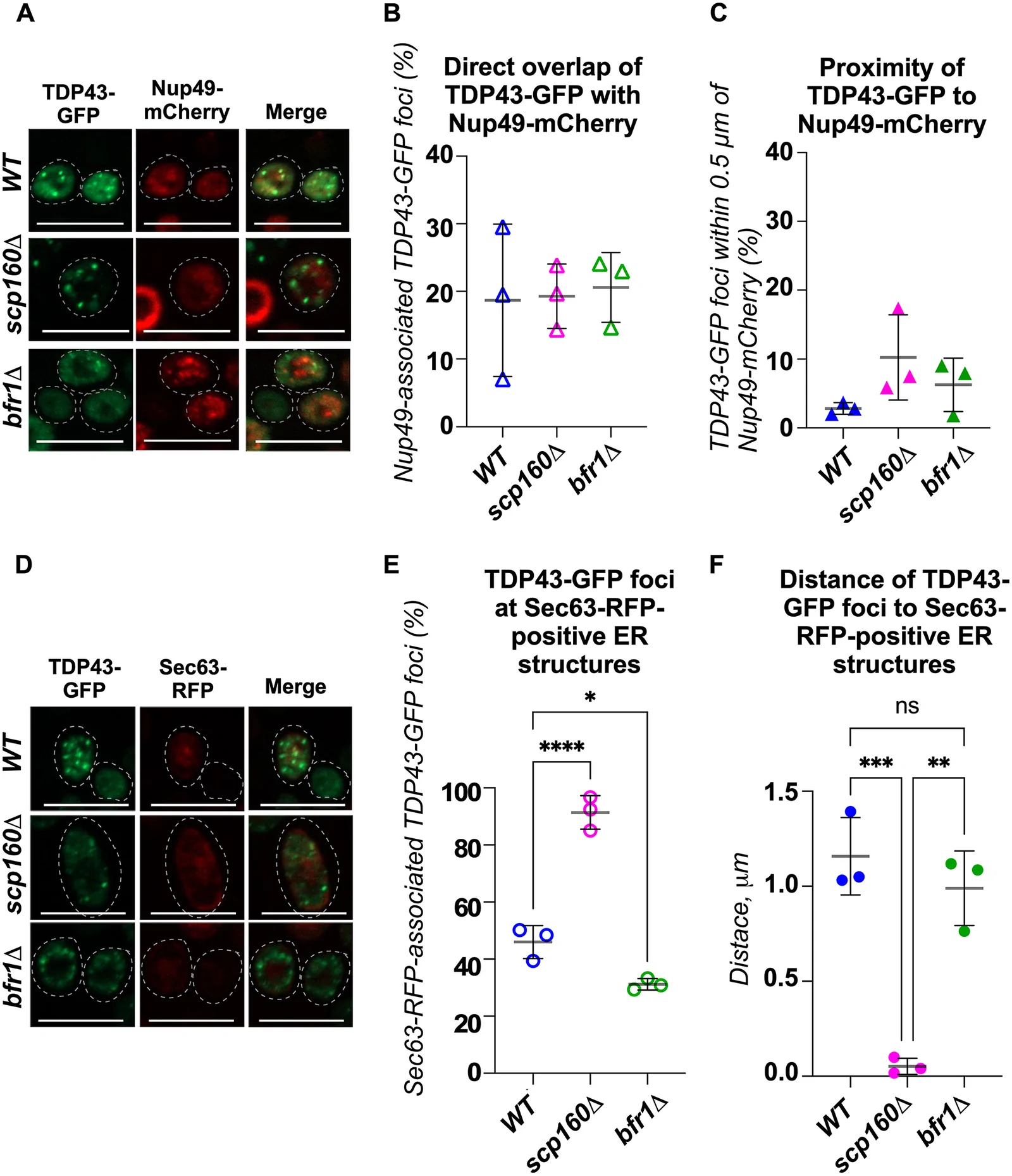
Loss of Scp160 alters the spatial association of TDP43-GFP foci with Sec63-positive ER structures but not Nup49-positive structures. **(A)** Representative fluorescence microscopy images of *WT*, *scp160* Δ , and *bfr1* Δ cells co-expressing TDP43-GFP and the nuclear pore marker Nup49-mCherry. TDP43-GFP, Nup49-mCherry, merged fluorescence, and merged fluorescence overlaid with bright-field (BF) images are shown. Dashed outlines indicate cell boundaries. Scale bars: 10 μ m. **(B)***Direct association of TDP43-GFP foci with Nup49-mCherry-positive structures.* TDP43-GFP foci were identified independently from the GFP channel and scored as Nup49-associated when they directly overlapped the Nup49-mCherry-positive signal. For each independent culture, the percentage of Nup49-associated TDP43-GFP foci was calculated relative to the total number of detected TDP43-GFP foci. Each symbol represents one independent biological culture; data are mean ± SD, *n* = 3 cultures per genotype. Differences were assessed by ordinary one-way ANOVA followed by Dunnett’s multiple-comparisons test using *WT* as the control. No significant genotype effect was detected, F(2,6) = 0.04765, *P* = 0.9538. Neither *scp160* Δ nor *bfr1* Δ differed significantly from *WT* (adjusted *P* = 0.9932 and 0.9363, respectively). **(C)***Proximity of TDP43-GFP foci to Nup49-mCherry-positive structures.* For each detected TDP43-GFP foci, the shortest distance to the nearest Nup49-mCherry-positive signal was determined. A predefined distance threshold of 0.5 μ m was used to classify foci as Nup49-proximal; foci located within 0.5 μ m of a Nup49-positive structure were considered proximal. For each independent culture, the percentage of Nup49-proximal TDP43-GFP foci was calculated relative to the total number of detected foci. Each symbol represents one independent biological culture; data are mean ± SD, *n* = 3 cultures per genotype. Differences were assessed by ordinary one-way ANOVA followed by Tukey’s multiple-comparisons test. The overall genotype effect was not significant, F(2,6) = 2.283, *P* = 0.1831. No significant differences were detected between *WT* and *scp160* Δ (adjusted *P* = 0.1626), *WT* and *bfr1* Δ (adjusted *P* = 0.6083), or *scp160* Δ and *bfr1* Δ (adjusted *P* = 0.5248). **(D)** Representative fluorescence microscopy images of *WT*, *scp160* Δ , and *bfr1* Δ cells co-expressing TDP43-GFP and the ER marker Sec63-RFP. TDP43-GFP, Sec63-RFP, merged fluorescence, and merged fluorescence overlaid with bright-field (BF) images are shown. Dashed outlines indicate cell boundaries. Scale bars: 10 μ m. **(E)***Percentage of TDP43-GFP foci associated with Sec63-RFP-positive ER structures.* TDP43-GFP foci were identified independently from the GFP channel, and Sec63-positive regions were defined independently from the RFP channel. Sec63-positive regions were defined from the upper 10% of background-corrected, field-relative RFP signal, and the resulting mask was expanded by one pixel to accommodate minor inter-channel registration differences. TDP43-GFP foci were classified as Sec63-associated when at least 20% of the GFP-defined foci area overlapped the Sec63-RFP-positive mask. For each independent culture, the percentage of Sec63-associated foci was calculated relative to the total number of detected TDP43-GFP foci. Each symbol represents one independent biological culture; data are mean ± SD, *n* = 3 cultures per genotype. Differences were assessed by ordinary one-way ANOVA followed by Dunnett’s multiple-comparisons test using *WT* as the control. The overall genotype effect was significant, *F*(2, 6) = 122.52, *P* < 0.0001. The percentage of Sec63-associated TDP43-GFP foci was strongly increased in *scp160* Δ cells relative to *WT* (adjusted *P* < 0.0001) and was modestly reduced in *bfr1* Δ cells relative to *WT* (adjusted *P* = 0.0178). **(F)***Distance of TDP43-GFP foci to the nearest Sec63-RFP-positive ER structure.* For each detected TDP43-GFP foci, the shortest Euclidean distance from the center (centroid) of the foci to the nearest Sec63-RFP-positive region was determined and converted to micrometers using the physical pixel dimensions stored in the OME metadata. Measurements from the two microscopy fields belonging to the same independent biological culture were pooled, and the mean center-to-Sec63 distance was used as the biological-replicate value. Each symbol represents one independent culture; data are mean ± SD, *n* = 3 cultures per genotype. Differences were assessed by ordinary one-way ANOVA followed by Tukey’s multiple-comparisons test. The overall genotype effect was significant, *F*(2, 6) = 39.02, *P* = 0.0004. TDP43-GFP foci were significantly closer to Sec63-positive ER structures in *scp160* Δ cells than in *WT* (adjusted *P* = 0.0004), whereas *bfr1* Δ did not differ significantly from *WT* (adjusted *P* = 0.4703). The difference between *scp160* Δ and *bfr1* Δ was also significant (adjusted *P* = 0.0011).

In *WT* cells, approximately 46% of TDP43-GFP foci were associated with Sec63-positive ER regions. This fraction increased to approximately 91% in *scp160* Δ cells, with the large majority of detectable foci meeting the Sec63-association criterion (Fig. 7**E**). In contrast, *bfr1* Δ cells showed a more modest but significant reduction in Sec63-associated foci, to approximately 31% (Fig. 7**E**). A similar pattern was observed in the distance analysis: TDP43-GFP foci in *scp160* Δ cells were markedly closer to the nearest Sec63-positive region than in *WT* or *bfr1* Δ cells. In contrast, the mean center-to-Sec63 distance in *bfr1* Δ cells did not differ significantly from *WT* (Fig. 7**F**).

Taken together, these analyses did not reveal significant genotype-dependent changes in TDP43-GFP association with mitochondria, Atg8-positive structures, or Nup49-positive nuclear-pore structures. In contrast, a pronounced spatial phenotype was observed for Sec63-positive ER regions in *scp160* Δ cells with both increased Sec63 association and reduced distance to Sec63-positive regions. *bfr1* Δ cells did not reproduce this pattern, despite their shared suppression of prominent TDP43-GFP aggregation. These findings suggest that the contributions of Scp160 and Bfr1 to TDP43 organization are not fully overlapping.

## DISCUSSION

In this study, we show that deletion of *SCP160* or *BFR1* suppresses TDP43-GFP toxicity without reducing steady-state reporter abundance and shifts TDP43-GFP away from prominent aggregate-like foci (Figs. 1 and 2). A similar protective phenotype was observed in *asc1* Δ cells, consistent with the recently reported effect of *ASC1* deletion on TDP-43 toxicity 26. Importantly, the effects of these deletions extended beyond TDP43: severe Htt103Q-GFP aggregates and VHL-GFP foci were also strongly reduced, indicating a broader influence on the intracellular organization of aggregation-prone and misfolded proteins (Figs. 3–5). At the same time, *scp160* Δ cells displayed a distinct spatial phenotype in which residual TDP43-GFP foci were markedly shifted toward Sec63-positive ER structures (Fig. 7**D–F**; Fig. S6). Together, these findings identify Scp160 and Bfr1 as factors that influence whether aggregation-prone proteins remain relatively dispersed or become concentrated into prominent intracellular structures, while also revealing a specific connection between Scp160 and the spatial organization of TDP43 relative to Sec63-positive ER regions.

### Scp160, Bfr1, and Asc1 influence the partitioning of misfolded proteins into visible assemblies

Across three distinct reporter systems, deletion of *SCP160*, *BFR1*, or *ASC1* consistently reduced the formation of prominent intracellular assemblies, while reporter abundance did not parallel the assembly phenotype. For TDP43-GFP, this redistribution was accompanied by improved growth despite comparable steady-state protein abundance, demonstrating that suppression of TDP-43 toxicity in the deletion strains does not require reduced reporter accumulation. These findings identify Scp160, Bfr1, and Asc1 as factors that influence the intracellular partitioning of aggregation-prone and misfolded proteins. The relationship between TDP-43 aggregation and toxicity nevertheless remains complex. Genetic suppression of TDP-43 toxicity can occur without reduced TDP-43 abundance while substantially altering its cytoplasmic organization, as shown for deletion of *DBR1* 14. Moreover, deep mutational analysis of the TDP-43 prion-like domain demonstrated that aggregate abundance alone does not predict toxicity, with larger, relatively immobile assemblies in some cases associated with reduced toxicity 15. Thus, although these previous studies indicate that the relationship between TDP-43 aggregation and toxicity is not straightforward, the close qualitative correspondence between reduced severe TDP43-GFP aggregation and improved growth in our deletion strains (Figs. 1**E** and 2**D**) suggests that altered TDP43 assembly contributes to the observed toxicity phenotype, while not establishing that the prominent aggregates in *WT* cells are themselves toxic.

The *asc1* Δ phenotype provides an important independent comparison that reinforces this broader relationship between visible TDP43 organization and toxicity. Park and colleagues similarly found that deletion of *ASC1* suppresses TDP-43 toxicity and produces smaller TDP43 foci in yeast and linked this protection to enhanced noncanonical autophagy 26. In our experiments, TDP43-GFP association with Atg8-positive structures was comparable in *WT*, *scp160* Δ , and *bfr1* Δ cells (Fig. S4), which raises the possibility that Scp160 and Bfr1 influence TDP43 toxicity through mechanisms that differ, at least in part, from the noncanonical autophagy pathway engaged in *asc1* Δ cells. However, more detailed analysis of autophagic flux and related autophagy pathways in *scp160* Δ and *bfr1* Δ cells will be needed to test this possibility.

The Htt103Q-GFP experiments demonstrate that the phenotype is not restricted to TDP43. Strong suppression of severe Htt103Q-GFP aggregation in *scp160* Δ is consistent with previous work showing that Scp160 promotes aggregation of polyglutamine reporters and endogenous Q/N-rich proteins 22. Earlier genetic screens also identified *bfr1* Δ as a suppressor of Htt103Q-mediated toxicity and recovered *SCP160*, *BFR1*, and *ASC1* among genes that influence polyQ-induced growth inhibition 33, 34. Our results extend these observations by showing that deletion of all three genes shifts Htt103Q-GFP away from the severe-aggregate state. Notably, although *BFR1* deletion was previously identified as a suppressor of Htt103Q-mediated toxicity 34, the effect of *BFR1* deletion on Htt103Q aggregation had not been directly characterized. Our data therefore provide new evidence that loss of Bfr1 substantially reduces severe Htt103Q-GFP aggregate formation. Importantly, this phenotype did not parallel reporter abundance: *scp160* Δ cells accumulated abundant Htt103Q-GFP while almost completely lacking severe inclusions (Fig. 3).

The VHL-GFP experiments extend this relationship to a biochemically distinct misfolded substrate. Folding-defective VHL is comparatively soluble, is handled by cytosolic chaperone systems, and is normally targeted for proteasomal degradation, but can accumulate in organized protein-quality-control assemblies when degradation becomes limiting 35–38. VHL-GFP foci were strongly suppressed in *scp160* Δ , *bfr1* Δ , and *asc1* Δ cells even following proteasome inhibition (Figs. 4 and 5). In *scp160* Δ and *bfr1* Δ , this occurred without reduced reporter abundance, whereas *asc1* Δ cells accumulated markedly elevated VHL-GFP while the reporter remained predominantly diffuse. Across three distinct substrates, reporter abundance therefore did not predict the formation of visible assemblies. We favor the interpretation that Scp160, Bfr1, and Asc1 influence the intracellular partitioning of misfolded proteins into visible assemblies, while the pathways producing this shared phenotype may differ among the mutants.

ThT staining provided an additional distinction between TDP43 and Htt103Q assemblies. TDP43-YFP foci lacked detectable ThT-specific enrichment in *WT*, *scp160* Δ , and *bfr1* Δ cells, whereas Htt103Q-YFP inclusions were clearly ThT-reactive (Fig. 6; Fig. S2), consistent with previous reports of ThT-negative TDP-43 aggregates and yeast TDP43 foci 31, 41. These results indicate that the altered morphology of TDP43 foci in *scp160* Δ and *bfr1* Δ cells is not accompanied by detectable acquisition of ThT-reactive amyloid-like organization. More broadly, they underscore that changes in visible aggregate morphology do not necessarily reflect conversion to an amyloid-like state and should be interpreted together with complementary structural and spatial measurements.

### Deletion of *SCP160* alters the spatial organization of TDP43 at the ER

Among the localization analyses, the most pronounced genotype-specific difference was observed at Sec63-positive ER regions. In *scp160* Δ cells, residual TDP43-GFP foci showed a marked spatial shift toward these regions: most detectable foci were Sec63-associated, and the mean distance to the nearest Sec63-positive region was substantially reduced relative to *WT,*while*bfr1* Δ cells showed a different pattern (Fig. 7**D–F**; Fig. S6). Direct Sec63 association was modestly but significantly reduced relative to *WT*, whereas the mean center-to-Sec63 distance did not differ significantly from *WT*. Thus, although deletion of *SCP160* and *BFR1* produced similar suppression of prominent TDP43-GFP aggregation, the two mutants were distinguishable in their spatial relationship to Sec63-positive ER regions. These findings suggest that the contributions of Scp160 and Bfr1 to TDP43 organization are not fully overlapping.

In contrast, TDP43-GFP association with MitoTracker-positive regions, Atg8-mCherry-positive structures, and Nup49-mCherry-positive nuclear-pore structures did not differ significantly among *WT*, *scp160* Δ , and *bfr1* Δ cells (Fig. 7**A–C**; Figs. S3-S5). The absence of corresponding changes at these markers argues against a generalized redistribution of TDP43 among multiple intracellular compartments and further emphasizes the selectivity of the Sec63-associated phenotype.

The ER-associated redistribution is particularly notable because Scp160 associates with polysomes and is enriched at the nuclear envelope and ER, including ER-associated translating ribosomes 18, 53, while Bfr1 participates in polysome-associated mRNP complexes with Scp160 and has ER-linked functions 23, 24. Spatial sequestration is also an established component of protein quality control 35, 45, 46, and TDP-43 assemblies associated with ER-exit sites have recently been described in mammalian cells 42. More recently, an ER-anchored isoform of the deubiquitinase USP19 was shown to promote aggregation of TDP-43 C-terminal fragments and couple TDP-43 proteostasis to ER stress in mammalian models 54. Together, these observations place the ER at a potentially important intersection between mRNP-associated regulation and the cellular handling of aggregation-prone proteins. In this context, the pronounced Sec63-associated shift observed specifically in *scp160* Δ cells (Fig. 7**D–F**, Fig. S6) raises the possibility that Scp160 contributes to the organization or handling of TDP43 assemblies within ER-associated protein-quality-control environments. Although our data do not establish the underlying mechanism or the precise ER subdomain involved, they identify a testable direction for future studies aimed at determining how Scp160-dependent pathways intersect with ER-associated protein quality control to influence TDP43 fate.

### A possible link between ribosome-associated mRNPs and protein assembly

The related phenotypes of *scp160* Δ and *bfr1* Δ , together with the similar phenotype of the *asc1* Δ comparator, are notable because all three proteins are functionally connected to translating ribosomes. Scp160 is a polysome-associated RNA-binding protein whose ribosome association depends in part on Asc1 27, a 40S-associated scaffold with established roles in translational fidelity and ribosome-associated quality-control responses to stalled ribosomes 55, 56. Bfr1 and Scp160 are components of overlapping polysome-associated mRNP complexes 24, 25, and both influence the expression or translational efficiency of selected mRNAs rather than acting as general translation factors 19, 23. These shared connections provide a plausible biological context for the broad changes in protein assembly observed here. At the same time, the distinct Sec63-associated phenotypes of *scp160* Δ and *bfr1* Δ indicate that their effects are not identical. This is consistent with Scp160 and Bfr1 functioning together in overlapping mRNP-associated processes while retaining separable cellular functions.

One possibility is that ribosome- and mRNP-associated organization influences the local environment in which newly synthesized aggregation-prone proteins are handled. The ribosome is an important platform for cotranslational folding, chaperone engagement, and protein quality control 20, 21, 57. Altered organization or localization of specific mRNPs could therefore influence whether newly synthesized proteins remain dispersed or enter visible assemblies. This model is consistent with the shared effects of *SCP160*, *BFR1*, and *ASC1* deletion across TDP43-GFP, Htt103Q-GFP, and VHL-GFP, while the ER-associated redistribution in *scp160* Δ suggests an additional spatial role for Scp160. The phenotypic relationships uncovered in this study thus provide a framework for testing how ribosome- and mRNP-associated organization influences the fate of aggregation-prone proteins. A logical next step will be to determine whether deletion of *SCP160* or *BFR1* alters reporter mRNA localization, ribosome occupancy, translation elongation, or cotranslational folding, thereby directly testing the proposed cotranslational model.

The evolutionary conservation of Scp160 extends the potential relevance of these findings beyond yeast. Scp160 belongs to the Vigilin family of multi-KH-domain RNA-binding proteins, and its human homolog HDLBP/Vigilin shares structural and functional features with the yeast protein 58. HDLBP is enriched at the cytoplasmic face of the ER, binds many ER-targeted mRNAs, and contributes to their translation; it has also been identified as a stress-granule-associated protein 59, 60. These parallels raise the possibility that conserved Vigilin-dependent pathways influence TDP-43 assembly or spatial partitioning, although a direct role for HDLBP in TDP-43 proteinopathy has not yet been established. From a therapeutic perspective, the relevant goal would not necessarily be to suppress TDP-43 aggregation indiscriminately, because different TDP-43 assembly states can have different relationships to toxicity 15, 16. Rather, determining whether HDLBP modulation can shift TDP-43 away from deleterious intracellular states may reveal new principles for manipulating TDP-43 proteostasis in ALS. Testing the effects of HDLBP depletion or modulation on TDP-43 assembly, localization, and toxicity in mammalian and neuronal systems will therefore be an important direction for future work.

### Limitations and future directions.

The localization survey narrows the most prominent genotype-dependent spatial phenotype to the ER. We did not detect significant differences in TDP43-GFP association with MitoTracker-positive mitochondrial regions (Fig. S3), Atg8-mCherry-positive structures (Fig. S4), or Nup49-mCherry-positive nuclear-pore structures (Fig. 7**A–C**; Fig. S5). These experiments did not reveal major differences in the localization parameters measured but do not exclude more specific functional changes involving these compartments. MitoTracker labeling can be influenced by mitochondrial membrane potential and other aspects of mitochondrial physiology 61, while Atg8 localization does not by itself measure autophagic flux 62, and Nup49 proximity does not directly assess nuclear import or export. More targeted analyses of mitochondrial function, autophagic flux, or nucleocytoplasmic transport may therefore reveal additional consequences of *SCP160* or *BFR1* deletion.

The ER-associated phenotype itself is supported by two complementary spatial measurements: *scp160* Δ cells showed both markedly increased TDP43-GFP association with Sec63-positive regions and a substantially reduced center-to-Sec63 distance (Fig. 7**E–F**). Sec63-RFP, however, labels the ER broadly and does not distinguish individual ER subdomains or ER-exit sites; in budding yeast, Sec63 has been used to delineate the general ER network, whereas COPII components such as Sec13 and Sec23 mark discrete ER-exit sites 63. We therefore do not conclude that the Sec63-associated TDP43 foci observed in *scp160* Δ cells correspond to the ERES-associated TDP-43 assemblies described in mammalian cells 42. ER abundance and geometry can also influence the probability of apparent overlap. In addition, TDP43-GFP foci differed visibly in morphology and apparent size among genotypes, which may influence both fractional-overlap and center-to-region distance measurements. Fractional overlap is sensitive to the size and shape of segmented objects, whereas center-to-region distance does not directly account for the spatial relationship between the boundaries of TDP43-GFP foci and Sec63-positive structures. This consideration may be particularly relevant to interpretation of the more modest change in Sec63 association observed in *bfr1* Δ cells. Object-based analyses that incorporate object volume, surface contact, and edge-to-edge distance would provide a more complete characterization of these spatial relationships 64. Nevertheless, the agreement between the overlap and distance measurements makes the spatial shift toward the ER well supported. Determining whether these foci associate with a particular ER subdomain will require direct ERES markers such as Sec13, Sec23, or Sec16 and higher-resolution three-dimensional imaging 63, 65, 66.

The *asc1* Δ results likewise receive support from their close agreement with the independently reported effects of *ASC1* deletion on TDP43 toxicity and foci morphology 26. An additional genetic consideration nevertheless applies to this strain because the *ASC1* intron encodes the U24 snoRNA/*SNR24*, which guides 2 ${}^{\prime }$ -*O*-methylation of 25S rRNA, and complete deletion of the *ASC1* locus therefore removes both Asc1 protein and the intronic snoRNA. Protein-null *ASC1* alleles that preserve *SNR24* expression have been developed specifically to distinguish these functions 67. The unusually strong effect of *asc1* Δ on VHL-GFP abundance (Fig. 5**C–D**) would therefore be particularly interesting to revisit using an Asc1 protein-specific allele that preserves *SNR24*.

Overall, our results support a model in which Scp160 and Bfr1 influence the intracellular partitioning of aggregation-prone and misfolded proteins into visible assemblies. Deletion of *SCP160* or *BFR1* suppresses prominent assembly formation across TDP43-GFP, Htt103Q-GFP, and VHL-GFP without a corresponding decrease in reporter abundance, while *SCP160* deletion produces an additional redistribution of residual TDP43-GFP foci toward Sec63-positive ER structures. These findings connect ribosome- and mRNP- associated factors to the spatial organization of misfolded proteins and identify Scp160 as a potential link between ER-associated RNA/ translation environments and TDP-43 organization.

## MATERIALS AND METHODS

### Yeast strains and culture

Wild-type *Saccharomyces cerevisiae* BY4742 (*MAT* α *his3* Δ *1 leu2* Δ *0 lys2* Δ *0 ura3* Δ *0*) and its derivative KanMX6 deletion strains (*scp160* Δ , *bfr1* Δ , and *asc1* Δ ) were obtained from Thermo Fisher Scientific. Cultures were grown in standard rich medium (YPDA: 1% yeast extract, 2% peptone, 2% glucose, supplemented with 10 mg/L adenine) or in the appropriate synthetic dropout medium containing glucose, or galactose as indicated. Unless otherwise specified, cultures were initiated from single colonies and grown overnight (approximately 16 h) at 30 ${}^{\circ }$ C in glucose-containing synthetic medium lacking the appropriate auxotrophic supplement (Ura − or Ura − /Leu − ). Prior to Gal induction, cells were washed twice with sterile water and resuspended in the appropriate galactose-containing selective medium to an OD ${}_{600}$ of approximately 0.4. Yeast transformations were performed using a standard lithium acetate/polyethylene glycol procedure.

### Chemicals and antibodies

Thioflavin T, > 95% (VWR, Cat. No. 76483-858); FM4-64 (Molecular Probes/Invitrogen, Thermo Fisher Scientific, Cat. No. T3166); MitoTracker Red (Molecular Probes, Cat. No. M7512); and bortezomib (VWR, Cat No. J60378-MA). Monoclonal anti-GFP antibodies were from Santa Cruz Biotechnology (sc-9996) and used at 800 ng/mL; mouse monoclonal anti- α -tubulin antibodies (12G10) were obtained from the Developmental Studies Hybridoma Bank (DSHB; University of Iowa, Iowa City, IA, USA) and used at a 1:1000 dilution; mouse monoclonal anti-FLAG antibodies (M2) were purchased from Sigma and used at 1 μ g/mL; anti-mouse IgG-HRP were from GE-Healthcare (NA931) and used at a dilution of 1:5000.

### Plasmids

The TDP43-GFP expression vector was based on the high-copy 2 μ plasmid pRS426 and expressed human TDP43-GFP under the control of the inducible *GAL1* promoter; the plasmid was obtained from Addgene (plasmid #27467, 5). The plasmid expressing TDP43-YFP under the control of the *GAL1* promoter, on a 2 μ backbone carrying *LEU2* as a selectable marker, was a kind gift from Dr. Katrina Cooper (Rowan-SOM). pYes-Htt103Q was a gift from Dr. Yury Chernoff (Georgia Institute of Technology) 68; pESC-LEU-GFP-VHL was obtained from Dr. Judit Frydman (Stanford University; Addgene #21053); the ER marker Sec63-RFP was expressed from plasmid pCG536, a kind gift from Dr. Campbell Gourlay (University of Kent, Canterbury, UK). The plasmid expressing Atg8 fused to mCherry was obtained from Dr. Daniel J. Klionsky (University of Michigan) and has been described previously 69. The nuclear pore marker Nup49-mCherry was expressed from a *LEU2*-based plasmid kindly provided by Dr. Kiran Madura (Rutgers Robert Wood Johnson Medical School). Plasmids expressing *WT* Rny1-FLAG and the glycosylation-defective Rny1-FLAG-W399R mutant were described previously 28.

### Cell viability and growth assays

*WT*, *scp160* Δ , *bfr1* Δ , and *asc1* Δ strains carrying the *P* ${}_{GAL1}$ -TDP43-GFP plasmid were grown overnight at 30 ${}^{\circ }$ C in glucose-containing synthetic complete medium lacking uracil (SC/Ura-). Cultures were diluted into fresh medium to an OD ${}_{600}$ of approximately 0.2 and grown for an additional 4 h at 30 ${}^{\circ }$ C. Cells were collected, washed, and aliquots containing 2 × 10 ${}^{6}$ cells were adjusted to a final volume of 200 μ L. Fivefold serial dilutions were prepared and spotted onto SC/Ura- agar plates containing glucose or galactose. Plates were incubated at 30 ${}^{\circ }$ C for 7 days before imaging.

Liquid-culture growth and TDP43-GFP fluorescence kinetics. *WT*, *scp160* Δ , *bfr1* Δ , and *asc1* Δ cells carrying the *P* ${}_{\mathrm{GAL1}}$ -TDP43-GFP plasmid were grown overnight at 30 ${}^{\circ }$ C in glucose-containing SC/Ura − medium. Cultures were collected, washed twice with sterile water, and resuspended in SC/Ura − medium containing either glucose or galactose to an initial OD ${}_{600}$ of 0.4. Aliquots of 200 μ L were transferred to black-walled, clear, flat-bottom 96-well plates and incubated at 30 ${}^{\circ }$ C with orbital shaking in a BioTek Synergy HT microplate reader. OD ${}_{600}$ and GFP fluorescence were measured at 30-min intervals for 36 h. GFP fluorescence was measured using excitation and emission wavelengths of 488 and 520 nm, respectively.

For analysis of TDP43-GFP accumulation, GFP fluorescence at each time point was normalized to the corresponding OD ${}_{600}$ value. Fluorescence/OD ${}_{600}$ values were then expressed relative to the initial measurement for the same well. The early fluorescence time course was used to evaluate reporter accumulation following galactose induction, and the 6-h values were used for endpoint statistical comparisons. The complete 36-h OD ${}_{600}$ dataset was used to generate the liquid-culture growth curves.

Three independent biological experiments were performed, with each culture measured in technical triplicate. Technical replicates were averaged within each biological experiment before calculation of mean growth and fluorescence values and before statistical analysis.

### Live-cell Thioflavin T staining

PMST buffer contained 20 mM potassium phosphate (pH 7.5), 1.2 M sorbitol, 13.1 mM HEPES (pH 7.6), 43.7 mM potassium acetate, 2.31 mM Mg ${}^{2+}$ (1.31 mM magnesium acetate plus 1 mM MgCl ${}_{2}$ ), and 0.1% Triton X-100.

Approximately one OD ${}_{600}$ unit of induced yeast cells was collected by centrifugation (3,000-5,000 × g, 3 min), washed once with PMST, and resuspended in PMST containing 50 μ g/mL Thioflavin T. Cells were incubated for 20 min at room temperature in the dark, washed once with PMST, and resuspended in 50 μ L PMST.

Cells were immobilized on Concanavalin A-coated microscopy glass plates 5 min before imaging. Live-cell fluorescence images were acquired immediately using bright-field, YFP (TDP-43-YFP; Htt103Q-YFP), and CFP (Thioflavin T) channels with identical acquisition settings for the Thioflavin T channel across all strains and experimental conditions.

### FM4-64 staining of vacuoles

For visualization of vacuolar membranes, 4.5 h-Gal-induced yeast cells were incubated with 40 μ M FM4-64 (Molecular Probes) for 15 min at 30 ${}^{\circ }$ C. Cells were then collected, transferred to fresh SC-Gal/Ura- medium without dye, and chased for 1 h 15 min at 30 ${}^{\circ }$ C. Following the chase, cells were immobilized on Concanavalin A-coated microscopy glass and imaged by fluorescence microscopy. The staining procedure was adapted from our previously described protocol 28.

For visualization of mitochondria 70, induced cells were incubated with 100 nM MitoTracker Red CMXRos (Molecular Probes/Life Technologies, Cat. No. M7512) for 30 min at 30 ${}^{\circ }$ C. Cells were then washed to remove excess dye, immobilized on Concanavalin A-coated microscopy glass, and imaged immediately by fluorescence microscopy.

### Cell lysis and Western blotting

Cell lysis and Western blotting were done as previously described 71. In brief, yeast cell pellets were resuspended in RIPA buffer (50 mM Tris-HCl (pH 8.0), 150 mM NaCl, 0.1% SDS, 1% Nonidet P-40, 0.5% deoxycholic acid, 50 mM NaF, 40 mM DL- α -glycerophosphate, 30 mM Na ${}_{3}$ P ${}_{2}$ O ${}_{7}$ , and 1 mM Na ${}_{3}$ VO ${}_{4}$ ), supplemented with the protease inhibitor cocktail (Pierce) and 1 mM PMSF. Acid-washed glass beads (Sigma, 225-600 μ m) were added to the cell suspensions, and cells were disrupted by vortexing for 30 min at 4 ${}^{\circ }$ C; lysates were clarified by centrifugation, and protein concentrations were determined using the Bradford assay (Bio-Rad). For Western blotting, 5 μ g of total protein per sample was resolved on 10% SDS-polyacrylamide gels and transferred to nitrocellulose membranes (GE). Membranes were probed with the indicated primary antibodies followed by HRP-conjugated secondary antibodies. Protein bands were detected by enhanced chemiluminescence using ECL Prime Western Blotting Detection Reagent and imaged using a Cytiva chemiluminescence imaging system. Band intensities were quantified using ImageQuant software. GFP-tagged proteins were normalized to the corresponding α -tubulin signal within each lane for quantitative analyses.

### Fluorescence microscopy and image analysis

#### Fluorescence microscopy

Images were acquired using an inverted Zeiss Observer Z1 fluorescence microscope equipped with an ApoTome 2 module, an AxioCam MRm camera, and a 63 × oil-immersion objective. GFP-, YFP-, CFP-, RFP-, and mCherry-associated fluorescence and fluorescent dyes were visualized using the appropriate filter sets. Images were acquired using Zeiss ZEN Imaging Software. Brightness and contrast were adjusted uniformly across images being compared, and no nonlinear image adjustments were applied.

Unless otherwise specified, cells carrying GAL1-driven fluorescent reporters were grown overnight at 30 ${}^{\circ }$ C in glucose-containing selective medium, collected, washed twice with sterile water, and transferred to the corresponding galactose-containing selective medium to initiate reporter expression. TDP43-GFP/YFP and Htt103Q-GFP/YFP were induced for 6 h before imaging. For VHL-GFP experiments, reporter expression was induced for 5 h, followed by treatment with 50 μ M bortezomib or an equivalent volume of DMSO for an additional 1 h before imaging. Cells carrying TDP43-GFP together with Sec63-RFP, Nup49-mCherry, or Atg8-mCherry were grown in SC/Ura-Leu- medium to maintain both plasmids.

For manual microscopy analyses, cells were identified using the corresponding bright-field images. Cells that were substantially cut by the image boundary, markedly out of focus, or could not be classified confidently were excluded from analysis.

#### Classification of fluorescent reporter localization

Localization of TDP43-GFP/YFP, Htt103Q-GFP/YFP, and VHL-GFP was classified using four mutually exclusive categories: no detectable GFP signal, diffuse GFP fluorescence without discrete foci, moderate aggregates or foci, and severe aggregates or foci. Moderate aggregates/foci were defined as one or more small fluorescent puncta, whereas severe aggregates/foci were defined as one large bright foci and/or multiple prominent foci within the same cell. Cells containing both small and large foci were assigned to the severe category.

#### Quantification of TDP43-GFP, Htt103Q-GFP, and VHL-GFP localization

1. For TDP43-GFP and Htt103Q-GFP, the percentage of cells in each localization category was calculated separately for each independent experiment relative to the total number of cells scored. The percentage of aggregate-positive cells was calculated as the number of cells containing moderate or severe aggregates divided by the total number of cells scored and multiplied by 100. The percentage of cells containing severe aggregates was calculated in the same manner using the severe-aggregate category alone.

2. For VHL-GFP, localization-class percentages were calculated relative to the total number of cells scored. Because a substantial fraction of cells in some conditions lacked detectable GFP signal, foci formation was additionally evaluated among GFP-positive cells only. The percentage of GFP-positive cells containing VHL-GFP foci was calculated as the number of GFP-positive cells containing moderate or severe foci divided by the total number of GFP-positive cells and multiplied by 100.

#### Quantification of ThT-channel signal at TDP43-YFP foci

Cells expressing TDP43-YFP were imaged in the YFP and CFP channels, with the CFP channel used to detect Thioflavin T (ThT)-associated fluorescence. Parallel samples not exposed to ThT were imaged using identical acquisition settings and served as negative controls. Htt103Q-YFP was used as a positive control for detection of ThT-reactive inclusions.

Raw fluorescence images were exported as OME-TIFF files without adjustment of fluorescence intensities. The five raw structured-illumination phase frames for each fluorescence channel were averaged to generate a single image for quantitative analysis. OME-TIFF files were read in Python using Pillow, and image processing, segmentation, and fluorescence measurements were performed using scikit-image, NumPy, and SciPy 72. The custom Python code used for quantitative analysis of TDP43-YFP and ThT fluorescence microscopy images is available through Zenodo, DOI: doi:10.5281/zenodo.22134077. TDP43-YFP foci were identified independently from the YFP channel, and CFP-channel fluorescence was subsequently measured at the corresponding YFP-defined foci.

For each YFP-defined TDP43 foci, mean CFP-channel intensity within the foci was compared with CFP-channel intensity in a surrounding local cellular reference region. Local CFP/ThT enrichment was expressed as the ratio of CFP intensity at the foci to CFP intensity in the surrounding reference region. A ratio of 1 therefore indicated no local enrichment, whereas values greater than 1 indicated increased CFP-channel fluorescence at the TDP43-YFP foci. The median foci-level enrichment ratio for each analyzed image field was calculated for subsequent statistical analysis.

#### Quantification of TDP43-GFP association with subcellular markers

Cells expressing TDP43-GFP together with Sec63-RFP, Nup49-mCherry, or Atg8-mCherry, or cells stained with MitoTracker Red, were analyzed to determine the spatial relationship of TDP43-GFP with the ER, nuclear pore-associated structures, autophagy-associated structures, and mitochondria, respectively. Raw fluorescence images were exported as OME-TIFF files without adjustment of fluorescence intensities and analyzed using custom Python scripts implemented with tifffile**,** scikit-image, NumPy, and SciPy. GFP and red-fluorescence channels were analyzed independently so that identification of TDP43-GFP foci was not influenced by the intensity or distribution of the corresponding organelle marker. The custom Python code used for Sec63-RFP segmentation, TDP43-GFP/Sec63-RFP association, and distance analyses is publicly available through Zenodo (DOI: doi:10.5281/zenodo.22135305).

#### For Sec63-RFP analysis

TDP43-GFP foci and Sec63-positive ER regions were identified independently from the GFP and RFP channels, respectively. Sec63-positive regions were defined from the upper 10% of background-corrected, field-relative RFP signal, and the resulting mask was expanded by one pixel to accommodate minor inter-channel registration differences. TDP43-GFP foci were classified as Sec63-associated when at least 20% of the GFP-defined foci area overlapped the Sec63-RFP-positive mask. The percentage of Sec63-associated foci was calculated relative to the total number of detected TDP43-GFP foci. As a complementary measure of spatial association, the shortest Euclidean distance from the center (centroid) of each TDP43-GFP foci to the nearest Sec63-RFP-positive region was determined and converted to micrometers using the physical pixel dimensions stored in the OME metadata. Measurements from the two microscopy fields belonging to the same independent biological culture were pooled before calculation of the replicate-level percentage of Sec63-associated foci or mean center-to-Sec63 distance.

#### For Nup49-mCherry analysis

TDP43-GFP foci and Nup49-positive structures were identified independently. Direct association was defined as spatial overlap between TDP43-GFP foci and Nup49-mCherry-positive signal. In a complementary proximity analysis, the shortest distance from each TDP43-GFP foci to the nearest Nup49-positive structure was determined. A predefined distance threshold of 0.5 μ m was used to classify TDP43-GFP foci as Nup49-proximal. Measurements from microscopy fields belonging to the same independent biological culture were combined before calculation of replicate-level values.

#### For Atg8-mCherry analysis

TDP43-GFP foci and Atg8-mCherry-positive objects were identified independently. TDP43-GFP foci were classified as Atg8-associated when the GFP- and mCherry-defined objects spatially overlapped. The percentage of Atg8-associated foci was calculated relative to the total number of detected TDP43-GFP foci. Measurements from microscopy fields belonging to the same independent biological culture were combined before calculation of replicate-level values.

#### For mitochondrial analysis

MitoTracker Red-positive regions were identified independently from the red-fluorescence channel. Mean TDP43-GFP fluorescence within MitoTracker-positive regions was compared with GFP fluorescence in corresponding local non-mitochondrial reference regions. Mitochondrial enrichment was expressed as the ratio of GFP intensity within MitoTracker-positive regions to GFP intensity in the local non-mitochondrial reference region; a ratio of 1 indicated no preferential enrichment of TDP43-GFP at MitoTracker-positive regions. Measurements from microscopy fields belonging to the same independent biological culture were combined before calculation of replicate-level values.

For all subcellular-marker analyses, the independent biological culture, rather than individual cells, TDP43-GFP foci, or microscopy fields, was treated as the independent experimental unit.

### Statistical analyses

Statistical analyses were performed using GraphPad Prism 10. Unless otherwise indicated, each biological replicate was treated as an independent observation, and data are presented as mean ± SD with individual biological replicates shown as symbols. *P* values < 0.05 were considered statistically significant. Where multiple comparisons were performed, adjusted *P* values were reported. Significance was indicated as follows: ns, not significant; (^⁎^) *P* < 0.05; (^⁎⁎^) *P* < 0.01; (^⁎⁎⁎^) *P* < 0.001; and (^⁎⁎⁎⁎^) *P* < 0.0001.

TDP43-GFP abundance measured by immunoblotting and normalized to tubulin was compared among *WT*, *scp160* Δ , *bfr1* Δ , and *asc1* Δ strains using ordinary one-way ANOVA followed by Dunnett’s multiple-comparisons test with *WT* as the control. Percentages of total TDP43-GFP aggregate-positive cells and cells containing severe TDP43-GFP aggregates were analyzed using ordinary one-way ANOVA followed by Tukey’s multiple-comparisons test.

Percentages of cells containing severe Htt103Q-GFP aggregates and Htt103Q-GFP abundance normalized to tubulin were analyzed by ordinary one-way ANOVA followed by Tukey’s multiple-comparisons test.

VHL-GFP foci formation and VHL-GFP abundance were analyzed by ordinary two-way ANOVA with genotype and bortezomib treatment as factors, including the genotype × treatment interaction. For foci formation, bortezomib- and DMSO-treated cultures were compared within each genotype using Šídák’s multiple-comparisons test, and mutant strains were compared with *WT* within each treatment condition using Dunnett’s multiple-comparisons test. VHL-GFP abundance was compared among genotypes within each treatment condition using Tukey’s multiple-comparisons test. Localization distributions shown in stacked-bar graphs were descriptive; inferential analyses were performed using values calculated separately for each biological replicate.

ThT-channel enrichment at TDP43-YFP foci was analyzed by ordinary two-way ANOVA with genotype and ThT treatment as factors, including the genotype × treatment interaction. Tukey’s multiple-comparisons test was used for pairwise genotype comparisons within each treatment condition.

For Sec63-RFP analyses, percentages of Sec63-associated TDP43-GFP foci were compared among genotypes using ordinary one-way ANOVA followed by Dunnett’s multiple-comparisons test with *WT* as the control. Mean distances between TDP43-GFP foci and the nearest Sec63-RFP-positive ER structure were analyzed by ordinary one-way ANOVA followed by Tukey’s multiple-comparisons test. For Nup49-mCherry analyses, percentages of directly associated TDP43-GFP foci were analyzed by one-way ANOVA followed by Dunnett’s test, whereas percentages of foci located within the predefined 0.5- μ m proximity threshold were analyzed by one-way ANOVA followed by Tukey’s test.

Percentages of TDP43-GFP foci associated with Atg8-mCherry-positive structures and TDP43-GFP enrichment ratios at MitoTracker Red-positive mitochondrial regions were compared among genotypes using ordinary one-way ANOVA followed by Tukey’s multiple-comparisons test.

For microplate reader measurements, technical replicates were averaged within each biological experiment before analysis. Growth and fluorescence time courses were plotted descriptively, whereas the 6-h normalized fluorescence/OD ${}_{600}$ values were compared among genotypes using ordinary one-way ANOVA followed by Tukey’s multiple-comparisons test.

## ACKNWOLDEGMENTS

This study was supported by National Institutes of Health (NIH) grant R01GM114308 to N.S. We are grateful to Dr. Katrina Cooper (Rowan-Virtua University School of Osteopathic Medicine) for the TDP43-YFP plasmid, Dr. Yury Chernoff (Georgia Institute of Technology) for pYes-Htt103Q-GFP, Dr. Campbell Gourlay (University of Kent) for the Sec63-RFP plasmid, Dr. Daniel J. Klionsky (University of Michigan) for the Atg8-mCherry plasmid, and Dr. Kiran Madura (Rutgers Robert Wood Johnson Medical School) for the Nup49-mCherry plasmid. We also thank Francesca Frontera for assistance with fluorescence microscopy using the Zeiss Observer Z1.

## SUPPLEMENTAL MATERIAL

All supplemental data for this article are available online at http://microbialcell.com/researcharticles/2026a-castells-ballester-microbial-cell/. 

## CONFLICT OF INTEREST

The authors declare no conflicts of interest.

## ABBREVIATIONS

ALS – amyotrophic lateral sclerosis

BZ – bortezomib

ER – endoplasmic reticulum

GFP – green fluorescent protein

Htt – huntingtin

RFP – red fluorescent protein

TDP-43 – TAR DNA-binding protein 43

ThT – thioflavin T

VHL – von Hippel-Lindau

WT – wild type

YFP – yellow fluorescent protein
