# Supplementary material for: Scp160 deletion suppresses TDP-43 aggregation and toxicity in Saccharomyces cerevisiae
Source: Microb Cell. 2026 Oct 6;13:358–77. doi: 10.15698/mic2026.10.888 (PMC13659451; doi:10.15698/mic2026.10.888)
Supplement: Supplementary file 1 [file mic-13-358-s01.pdf]

Supplemental material

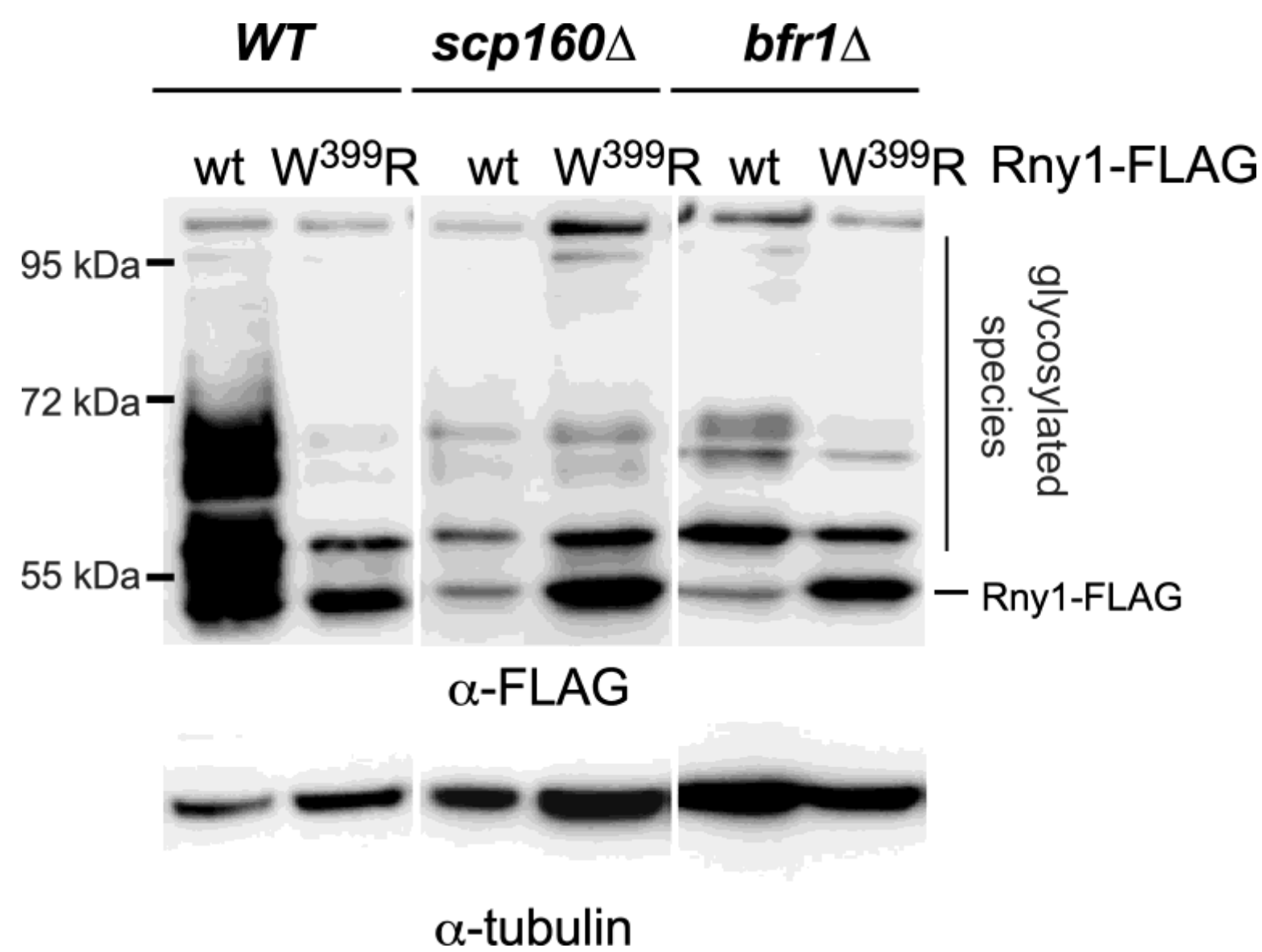

<u>Figure S1.</u> Rny1-FLAG expression in WT, scp160Δ, and bfr1Δ cells.

Immunoblot analysis of wild-type and W399R Rny1-FLAG in *WT, scp160Δ,* and *bfr1Δ* cells. Rny1-FLAG species were detected using anti-FLAG antibodies, and tubulin served as a loading control. Wild-type Rny1-FLAG migrated as multiple species, including heterogeneous higher-molecular-weight forms consistent with secretory-pathway glycosylation. The W399R mutant showed reduced accumulation of these heterogeneous glycosylated species. The positions of the major Rny1-FLAG species and glycosylated forms are indicated.

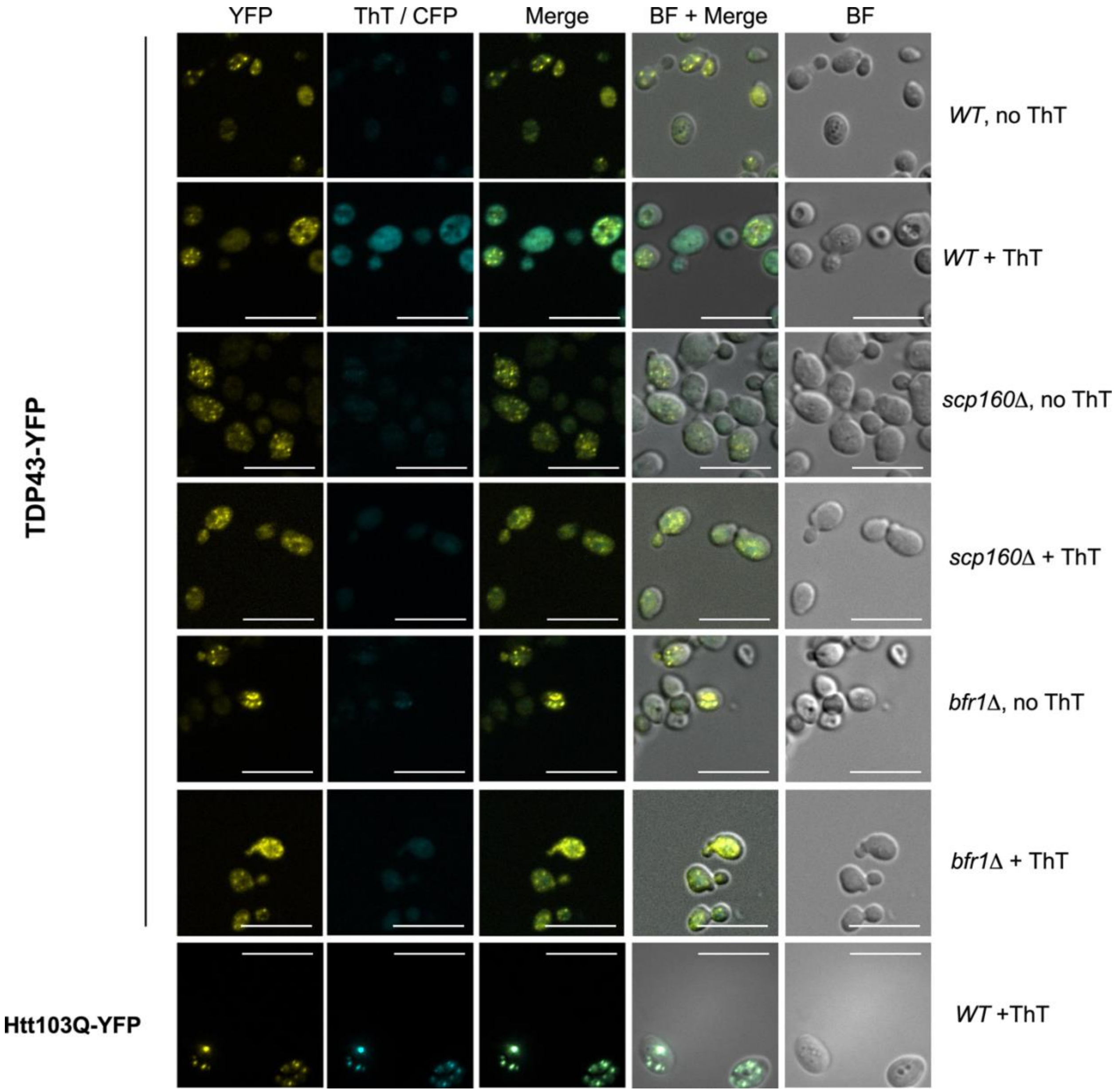

Figure S2. Extended fluorescence microscopy images of TDP43-YFP cells with and without Thioflavin T staining. Representative images of *WT*, *scp160Δ*, and *bfr1Δ* cells expressing TDP43-YFP in the absence or presence of Thioflavin T (ThT). TDP43-YFP fluorescence was detected in the YFP channel, and ThT-associated fluorescence was recorded in the CFP channel. YFP, ThT/CFP, merged fluorescence, BF + merge, and bright-field (BF) images are shown. *WT* cells expressing Htt103Q-YFP and stained with ThT are included as a positive control for detection of ThT-reactive inclusions. The corresponding quantitative analysis is shown in Figure 6B.

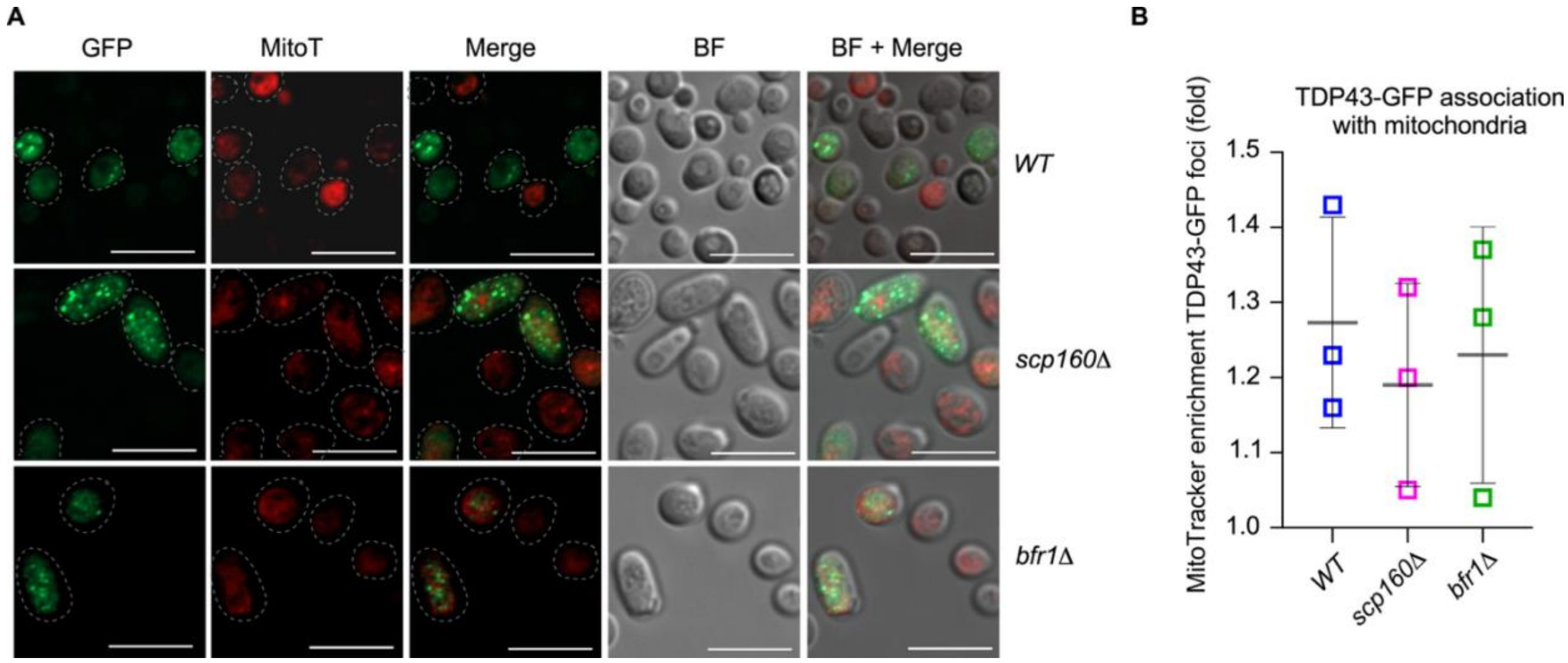

<u>Figure S3.</u> Figure S3. TDP43-GFP enrichment at MitoTracker Red-positive mitochondrial regions does not differ significantly among *WT, scp160Δ*, and *bfr1Δ* cells.

(A) Representative fluorescence microscopy images of *WT*, *scp160Δ*, and *bfr1Δ* cells expressing TDP43-GFP and stained with MitoTracker Red are shown together with the corresponding merged images. Dashed outlines indicate cell boundaries. Scale bars: 10 μm.

(B) TDP43-GFP fluorescence was quantified at MitoTracker Red-positive mitochondrial regions and compared with local non-mitochondrial background fluorescence to calculate fold enrichment of TDP43-GFP at MitoTracker Red-labeled regions. Each point represents an independent biological experiment, and horizontal lines indicate mean ± SD. Mean enrichment values were 1.273 for *WT*, 1.190 for *scp160Δ*, and 1.230 for *bfr1Δ*. No significant difference among strains was detected by ordinary one-way ANOVA, $F(2, 6) = 0.2332$, $P = 0.7988$. Tukey's multiple-comparisons test likewise showed no significant differences: *WT* versus *scp160Δ*, $P = 0.7817$; *WT* versus *bfr1Δ*, $P = 0.9336$; and *scp160Δ* versus *bfr1Δ*, $P = 0.9431$. Three independent biological experiments were analyzed for each strain.

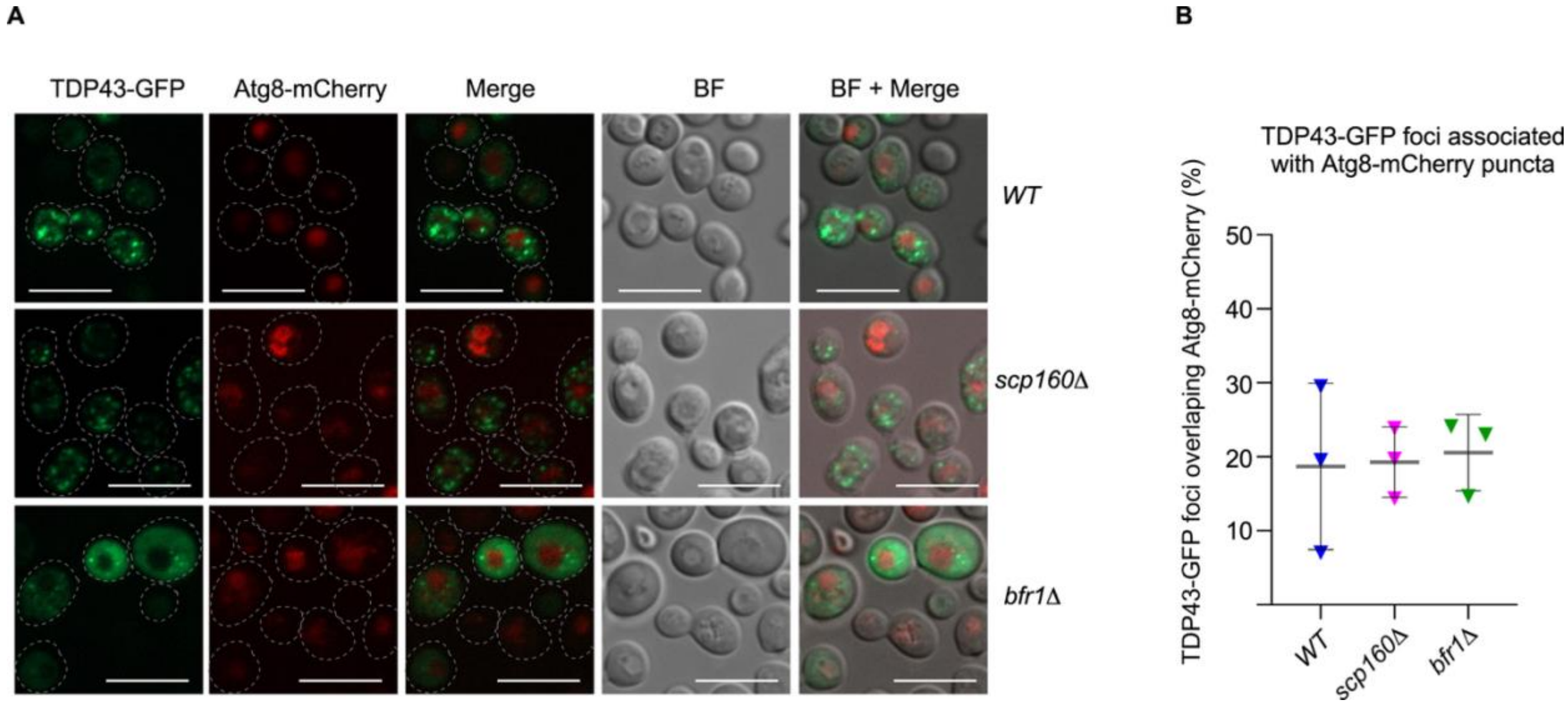

<u>Figure S4.</u> TDP43-GFP foci show similar association with Atg8-mCherry-positive structures in *WT, scp160Δ,* and *bfr1Δ* cells.

(A) Representative fluorescence microscopy images of WT, *scp160Δ*, and *bfr1Δ* cells co-expressing TDP43-GFP and Atg8-mCherry. TDP43-GFP, Atg8-mCherry, merged fluorescence, bright-field (BF), and BF + merge images are shown. Dashed outlines indicate cell boundaries. Scale bars: 10 µm.

(B) Quantification of the percentage of TDP43-GFP foci overlapping Atg8-mCherry-positive objects. For each analyzed field, discrete TDP43-GFP foci were identified and counted, and each focus was scored as overlapping when the GFP-positive object spatially intersected an Atg8-mCherry-positive object. The percentage of overlapping TDP43-GFP foci was calculated as: *(number of TDP43-GFP foci overlapping an Atg8-mCherry-positive object / total number of TDP43-GFP foci) × 100*. Measurements from microscopy fields belonging to the same independent culture were combined before calculation of replicate-level values. Each point represents one independent biological culture; n = 3 per strain. Data are shown as mean ± SD. Mean overlap was 18.69% for *WT*, 19.29% for *scp160Δ*, and 20.58% for *bfr1Δ*. No significant differences were detected among strains by ordinary one-way ANOVA followed by Tukey's multiple-comparisons test. Tukey-adjusted *P* values were *WT* vs. *scp160Δ*, *P* = 0.9949; *WT* vs. *bfr1Δ*, *P* = 0.9513; *scp160Δ* vs. *bfr1Δ, P* = 0.9770.

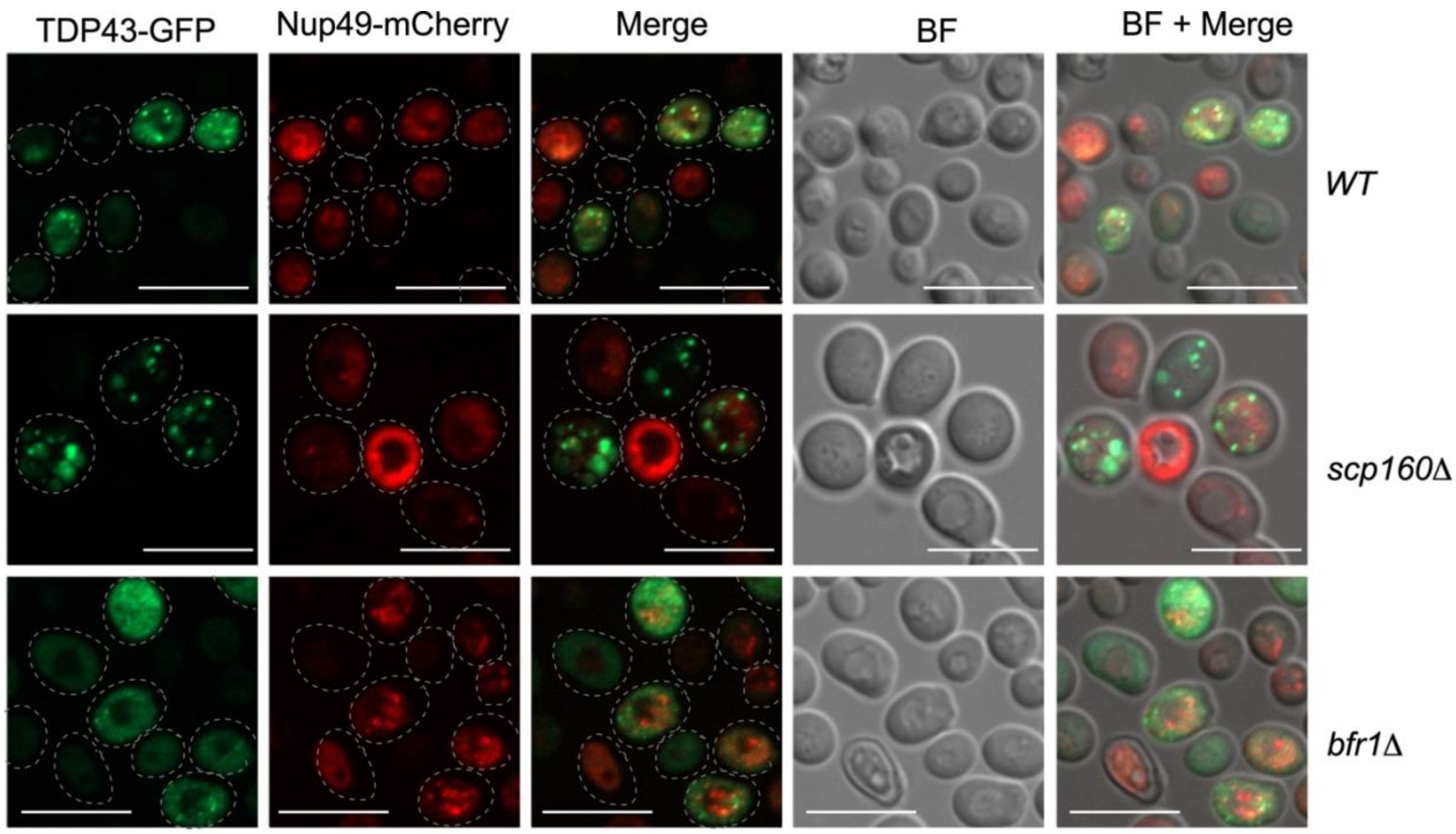

<u>Figure S5.</u> Extended fluorescence microscopy images of *WT, scp160Δ*, and *bfr1Δ* cells co-expressing TDP43-GFP and the nuclear pore/nuclear-envelope marker Nup49-mCherry. TDP43-GFP, Nup49-mCherry, merged fluorescence, bright-field (BF), and BF/fluorescence merged images are shown. Dashed outlines indicate cell boundaries. Scale bars: 10 µm. The corresponding quantitative analysis is shown in Figure 7B-C.

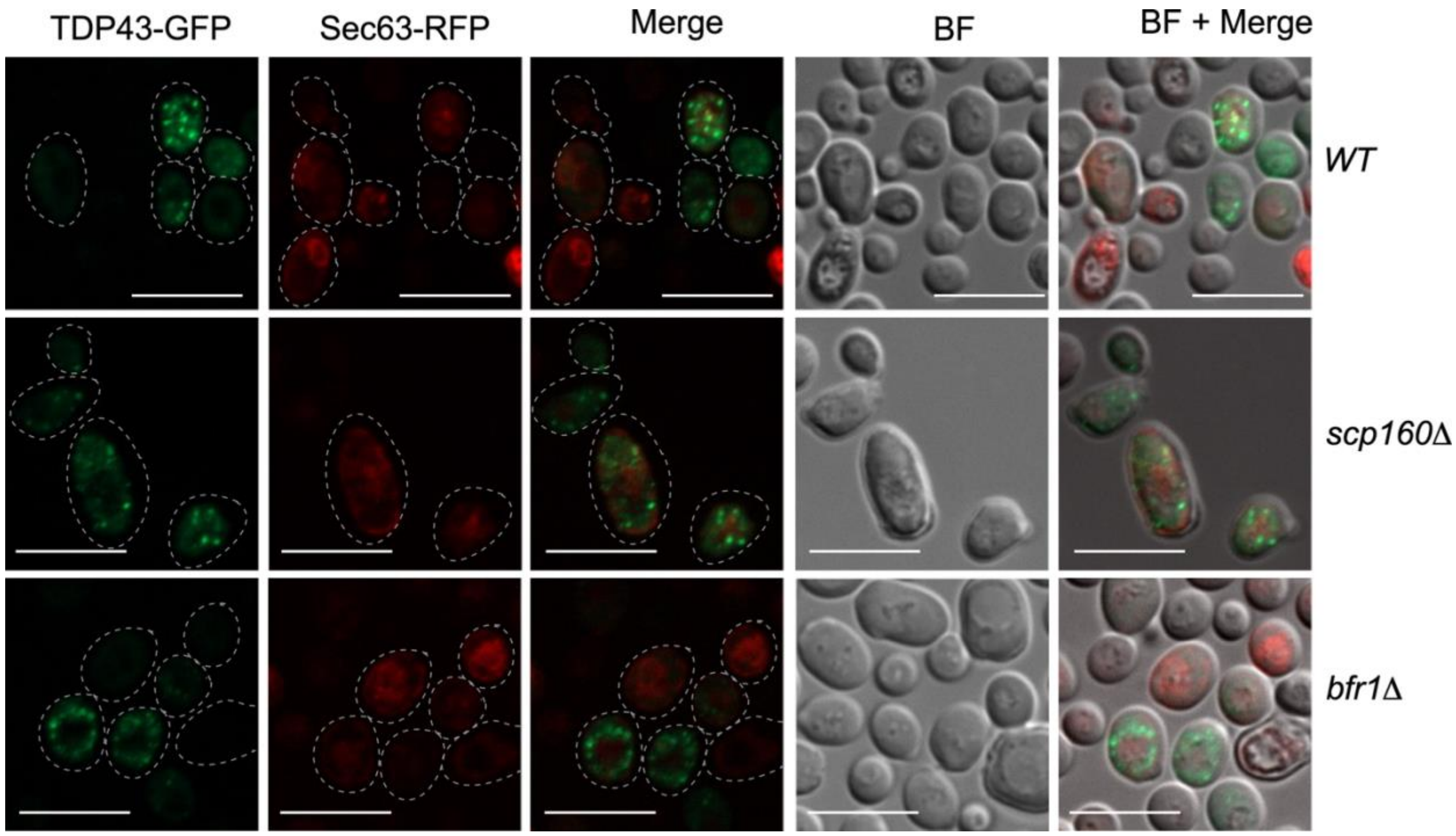

<u>Figure S6.</u> **Extended fluorescence microscopy images of *WT*, *scp160Δ*, and *bfr1Δ* cells co-expressing TDP43-GFP and the ER marker Sec63-RFP.** TDP43-GFP, Sec63-RFP, merged fluorescence, bright-field (BF), and BF plus merged fluorescence images are shown. Dashed outlines indicate cell boundaries. Scale bars: 10 µm. The corresponding quantitative analysis is shown in Figure 7E-F.
